# Supersensitive detection and discrimination of enantiomers by dorsal olfactory receptors: evidence for hierarchical odour coding

**DOI:** 10.1038/srep14073

**Published:** 2015-09-11

**Authors:** Takaaki Sato, Reiko Kobayakawa, Ko Kobayakawa, Makoto Emura, Shigeyoshi Itohara, Miwako Kizumi, Hiroshi Hamana, Akio Tsuboi, Junzo Hirono

**Affiliations:** 1 Biomedical Research Institute, National Institute of Advanced Industrial Science and Technology, Hyogo 661-0974, Japan; 2 Institute of Biomedical Science, Kansai Medical University, Osaka 573-1010, Japan; 3 Takasago International Corporation, Kanagawa 254-0073, Japan; 4 Laboratory for Behavioral Genetics, Brain Science Institute, RIKEN, Saitama 351-0198, Japan; 5 Research Institute of Frontier Medicine, Nara Medical University, Nara 634-8521, Japan

## Abstract

Enantiomeric pairs of mirror-image molecular structures are difficult to resolve by instrumental analyses. The human olfactory system, however, discriminates (−)-wine lactone from its (+)-form rapidly within seconds. To gain insight into receptor coding of enantiomers, we compared behavioural detection and discrimination thresholds of wild-type mice with those of ΔD mice in which all dorsal olfactory receptors are genetically ablated. Surprisingly, wild-type mice displayed an exquisite “supersensitivity” to enantiomeric pairs of wine lactones and carvones. They were capable of supersensitive discrimination of enantiomers, consistent with their high detection sensitivity. In contrast, ΔD mice showed selective major loss of sensitivity to the (+)-enantiomers. The resulting 10^8^-fold differential sensitivity of ΔD mice to (−)- vs. (+)-wine lactone matched that observed in humans. This suggests that humans lack highly sensitive orthologous dorsal receptors for the (+)-enantiomer, similarly to ΔD mice. Moreover, ΔD mice showed >10^10^-fold reductions in enantiomer discrimination sensitivity compared to wild-type mice. ΔD mice detected one or both of the (−)- and (+)-enantiomers over a wide concentration range, but were unable to discriminate them. This “enantiomer odour discrimination paradox” indicates that the most sensitive dorsal receptors play a critical role in hierarchical odour coding for enantiomer identification.

Enantiomeric pairs are difficult to resolve by instrumental analyses because compounds with mirror-image molecular structures have almost identical physicochemical properties. The olfactory system, however, discriminates enantiomers sensitively and rapidly within seconds. The relevant molecular information is encoded peripherally via stereospecific ligand binding to olfactory receptors (ORs) and relayed to the brain for processing to generate odour percepts. The human olfactory system detects (−)- and (+)-enantiomers of carvones with nearly equal sensitivity^1^−^3^. In contrast, humans are 10^8^-fold more sensitive to (−)-wine lactone compared to its (+)-form^4^. This striking difference illustrates that the detection of certain odours depends critically on stereospecific molecular binding to some ORs. To better understand receptor coding of enantiomers, we analysed differences in enantiomer detection and discrimination between wild-type (WT) and ΔD mice, in which olfactory receptors in the dorsal zone of the olfactory epithelium are genetically ablated. Dorsal receptors may play special roles in odour coding and recognition. We previously showed that ΔD mice are able to detect a component of fox faeces, 2,5-dihydro-2,4,5-trimethylthiazoline (TMT), but cannot recognise it as a predator odour^5^.

In mice, each olfactory sensory neuron expresses 1 of ca. 1,000 different types of ORs^6^,^7^,^8^,^9^ that operate as independent coding channels. We previously estimated that enantiomers of carvone (at 100 μM) activated ca. 70 types of murine ORs with >80% overlap^10^. The high overlap at higher odourant concentrations suggested that a minority of the most sensitive receptors plays a key role in determining odour quality differences between enantiomers and enabling their discrimination. This led us to formulate a model of odour quality coding in which signals transduced by cognate receptors and relayed as inputs through segregated channels in the olfactory bulb^11^,^12^,^13^, are processed in the olfactory cortex to evoke ‘elemental’ perceived odour qualities. Such processing could be mediated by feedforward, feedback, and associative connections in the cortex^10^,^14^,^15^,^16^,^17^,^18^.

The sensory profile of an odour stimulus may include several distinct elemental odours if multidimensional input is segmented through parallel pathways^19^. We proposed that elemental odours emerge hierarchically through a temporal coding scheme that prioritises the most sensitive, best-tuned receptors. These receptors would dominate the perceived odour qualities by relaying the earliest ascending signals to cortical targets, evoking specific elemental odours and recruiting feedforward inhibition to suppress competing odours evoked by OR inputs that are initially weaker and become stronger at later times. Our model predicts selective shifts in perceived odours by mutual inhibition when different stimuli are mixed to selectively shift the balance of best-tuned sensitive receptors. For example, TMT induces stress responses in mice when recognised as a predator odour^5^. The stress responses are reduced in different ways through feedforward inhibition when TMT is mixed with rose or hinokitiol odours, but not when TMT is mixed with (*S*)-(+)-carvone^20^,^21^.

Elemental odours encoded by subsets of orthologous ORs may be broadly conserved across species. Patterns of similarity among murine OR codes for 12 odourants resembled groupings of human percepts of the same odourant set^22^. Notably, three distinct subsets of murine ORs completely matched human odour percepts of vanilla, creamy and cinnamon, respectively. Here, we extended these findings by showing similarities of enantiomer odour detection sensitivity difference across humans and ΔD mice. This suggests that humans lack highly sensitive orthologous dorsal receptors for the (+)-enantiomer, similar to ΔD mice. Our comparison of behavioural discrimination thresholds with detection thresholds in WT and ΔD mice indicates that the most sensitive dorsal receptors play a critical role in hierarchical odour coding for enantiomer identification.

## Results

**Figure 1:**
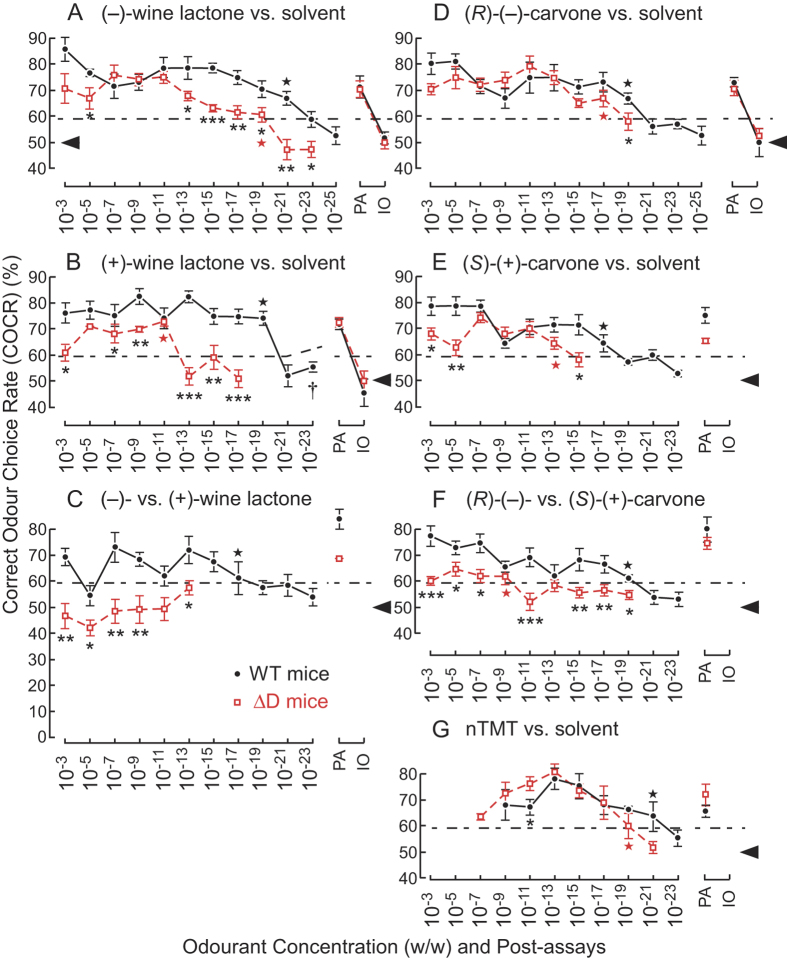
Behavioural difference in enantiomer detection and discrimination between WT and ΔD mice. Two alternative forced choice assays with odourants vs. solvent [di(propylene) glycol] or enantiomeric pairs of odourants were performed in a Y-maze. The correct odour choice rates (COCR) ± standard error of the mean (S.E.M.; 18 trials × 6 mice) are shown for wild-type (WT, black closed circles) and ΔD mice (red open squares). Tasks performed at thresholds are marked by the larger asterisks. A, Odour detection of (−)-wine lactone. Post-assays (PA): 10^−13^ (−)-wine lactone vs. solvent; identical odour (IO), 10^−13^ (−)-wine lactone vs. 10^−13^ (−)-wine lactone. B, Odour detection of (+)-wine lactone. PA: 10^−19^ (+)-wine lactone for WT mice; PA and IO, 10^−11^ and 10^−9^ (+)-wine lactone for ΔD mice, respectively. C, Odour discrimination between (−)- and (+)-wine lactone. PA: 10^−13^ (−)-wine lactone. D, Odour detection of (*R*)-(−)-carvone. PA: 10^−11^ and 10^−9^ (*R*)-(−)-carvone for WT and ΔD mice, respectively. E, Odour detection of (*S*)-(+)-carvone. PA: 10^−11^ (*S*)-(+)-carvone. F, Odour discrimination between (*R*)-(−)- and (*S*)-(+)-carvone. PA: 10^−11^ (*R*)-(−)-carvone. G, Odour detection of non-dihydrogenated TMT (nTMT). PA: 10^−21^ and 10^−17^ nTMT for WT and ΔD mice, respectively. The dashed lines indicate COCR significantly above chance performance [*P* = 0.05 for 108 or 54 (†) trials (6 and 3 mice, respectively), χ^2^-test]. Black arrowheads indicate chance levels (50%). Asterisks indicate concentrations at which the COCR for ΔD mice significantly differed from that of WT mice (*P < 0.05, **P < 0.01 and ***P < 0.001).

We conducted two alternative forced choice behavioural assays in a Y-maze to measure odourant detection thresholds of mice in a 100-fold dilution series. The Y-maze design (Supplementary Information Fig. S1) was motivated by the observation that moths and other insects sensitively navigate their way to scent sources along odour plumes^23^,^24^. Each mouse was trained to choose a target odour at the Y-junction and to run to the end of the arm to receive a water reward. Rewarded targets were: odourant solution vs. solvent (detection assay); and (−)- vs. (+)-enantiomer solution (discrimination assay). Detection threshold was defined as the lowest concentration of odourant solution for which the average correct odour choice rate (COCR) for the target odour was significantly higher than chance (>59.43%, *P* < 0.05 for 108 trials; χ^2^-test, Supplementary Information Table ST1). As expected, the performance of mice in detection tasks declined at lower odourant concentrations. Fig. 1 plots the downtrend of COCR with decreasing concentration. We focused on comparing threshold concentrations, where only the most sensitive ORs would be activated.

### Mice exhibit supersensitivity to wine lactones

We first examined sensitivity differences of mice to enantiomeric wine lactones. In humans, (−)-wine lactone, a trace compound of white wine, evokes a coconut-like sweet odour. We found that WT mice displayed a remarkable supersensitivity to both (−)- and (+)-wine lactones, with thresholds of 10^−21^ and 10^−19^ w/w, (10^−6^ and 10^−4^ ppq), respectively (larger asterisks in Fig. 1A,B, Table 1). Immediately after completing tests of each concentration series, we checked consistency of odour choice at one concentration (post-assays, PA), and also checked inability to select identical odours (IO) at the same concentration. Significant COCRs for targets in post-assays (10^−13^ and 10^−19^ w/w for (−)- and (+)-wine lactones, respectively), and no significant difference from chance in IO assays were always observed. Thus, mice remembered their odour choices around thresholds.

**Figure 2:**
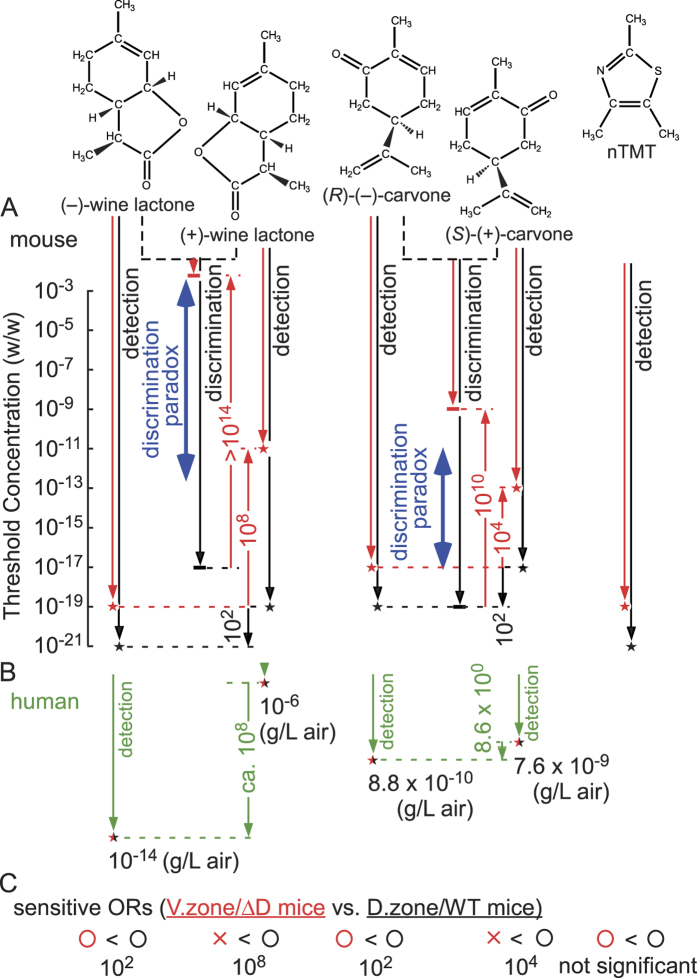
Comparison of detection and discrimination performance for wine lactone and carvone enantiomer pairs in WT and ΔD mice. (**A**) Plots of detection and discrimination range (arrows) and threshold (asterisks) for enantiomer pairs of odourants in WT mice (black plots) and ΔD mice (red plots) in the Y-maze. WT mice showed supersensitivity to both odourants with threshold concentrations, 10^−17^–10^−21^ w/w (10^−2^–10^−6^ ppq); ΔD mice showed 10^8^- and 10^4^-fold reductions in sensitivity to (+)-enantiomers of wine lactone and carvone, respectively, but only 10^2^-fold reductions in sensitivity to (−)-enantiomers. For wine lactones, the sensitivity difference between enantiomers was similar in humans and ΔD mice (10^8^-fold, green and red arrows between broken lines, respectively). However, for carvones, the differences in WT and ΔD mouse strains, and humans, were inconsistent (10^2^-, 10^4^- and 8.6-fold, black, red, and green arrows, respectively). Despite retention of high detection sensitivity to (−)-enantiomers, ΔD mice showed a >10^10^-fold reduction in discrimination sensitivity. This leads to an enantiomer odour discrimination paradox, in which ΔD mice detected one or both of the (−)- and (+)-enantiomers but could not discriminate them over a wide concentration range (blue arrows). (**B**) Detection thresholds in humans (black-red asterisks) as reported previously^3^,^4^. The differences in odourant detection thresholds are ca. 10^8^- and 8.6-fold for wine lactone and carvone enantiomers, respectively. (**C**) Inferred zonal distribution of sensitive olfactory receptors (ORs). The slightly higher sensitivity of WT mice and retention of high sensitivity in ΔD mice indicate the existence (O) of the most sensitive ORs for the target odourants in the dorsal (D.) and ventral (V.) zones of the olfactory epithelium. Decreases in sensitivity of ΔD mice indicate a lack (X) of the most sensitive ORs in the V. zone.

Compared to WT mice, detection sensitivity of ΔD mice was reduced 10^8^-fold for (+)-wine lactone (10^−11^ w/w), although only 100-fold for (−)-wine lactone (10^−19^ w/w) (Fig. 1A,B, Supplementary Information Table ST1). Again, consistency of odour choices was confirmed by post-assays. Since only ventral ORs are expressed in ΔD mice, our results imply that no ventral ORs are highly sensitive to (+)-wine lactone, although some are highly sensitive to (−)-wine lactone (Fig. 2C). The 10^8^-fold difference in sensitivity of ΔD mice to (−)- and (+)-wine lactones was the same as that observed in humans^4^ (Fig. 2A,B, Table 1). This suggested the possibility that the large difference in human sensitivity to these enantiomers is attributable to a lack of orthologous ORs of the murine dorsal zone that are most sensitive to (+)-wine lactone.

### Mice exhibit supersensitivity to carvones

For comparison, we also determined behavioural thresholds for carvone enantiomers. WT mice were supersensitive to both (*R*)-(−)- and (*S*)-(+)-carvones (10^−19^ and 10^−17^ w/w, respectively) (larger asterisks in Fig. 1D,E, Table 1). In ΔD mice, detection sensitivity was reduced 10^4^-fold for (*S*)-(+)-carvone, and 100-fold for (*R*)-(−)-carvone. These results indicated that the dorsal ORs most sensitive to (*S*)-(+)-carvone are 10^4^-fold more sensitive than the most sensitive ventral ORs (Fig. 2C). On the other hand, the dorsal ORs most sensitive to (*R*)-(−)-carvone are equally, or 10^2^-fold more sensitive than the most sensitive ventral ORs. The observed differences in sensitivities of ΔD and WT mice (10^4^- and 10^2^-fold, respectively) was much greater than the corresponding 8.6-fold sensitivity difference in humans^3^ (Fig. 2B, Table 1). Moreover, humans are roughly 10^5^-fold less sensitive to (*R*)-(−)-carvone than to (−)-wine lactone (Fig. 2B). These results indicate that humans express neither the orthologous murine ORs most sensitive to (*S*)-(+)-carvone, nor those of the both the dorsal and ventral ORs most sensitive to (*R*)-(−)-carvone. Humans express fewer than half the number of ORs found in mice. Absence of the most sensitive orthologous ORs could likely account for the much poorer detection and discrimination performance of humans compared to mice.

### Receptor coding is more sparse for wine lactones than carvones

Using Ca^2+^ imaging to profile odourant responses of olfactory sensory neurons (OSNs) of WT mice, we found that OR coding was sparser for wine lactone than carvones, i.e. wine lactone-sensitive ORs were about 3-fold less numerous than carvone-sensitive ORs (Supplementary Information Tables ST2, ST3). Sparse coding is consistent with the greater impact of dorsal OR ablation on behavioural detection thresholds of wine lactones. The largest subpopulations of the most sensitive ORs were those best-tuned to (−)-wine lactone or those with overlapping sensitivity to (−)-/(+)-wine lactones. These proportions held at all tested concentrations, except for (+)-wine lactone at 1 μM (Supplementary Information Table ST2, Fig. S2). Moreover, we did not observe (+)-wine lactone-sensitive ORs, except for one OR at the concentrations of <100 μM. These data are consistent with sparse coding of (+)-wine lactone by a small set of dorsal ORs with high sensitivity and selectivity.

### Sub-ppq level detection of general odourants

Supersensitivity of mice to carvone and wine lactone enantiomers may be surprising given that these compounds are usually regarded as general odourants. Extreme sensitivity is often associated with innate responses to semiochemicals such as allomones and pheromones^25^. The reported thresholds of WT and ΔD mice for TMT, an allomone of rodent predators, are equally low at 1.3 × 10^−8^ w/w (after correcting for volume), and much lower than for 2-methylbutyric acid (5.7 × 10^−5^ and 5.7 ×  × 10^−4^ w/w, respectively) in the habituation-dishabituation test^5^. Instead of TMT, we used non-dihydrogenated TMT (nTMT) to avoid potential problems of exposing WT mice to >2-weeks of repeated assays with the innate stressor TMT. We have observed supersensitive detection of nTMT in both WT mice (10^−21^ w/w) and ΔD mice (10^−19^ w/w) (larger asterisks in Fig. 1G, Table 1). Differences from the earlier data might be due either to our use of the Y-maze assay (vs. habituation-dishabituation assay), or to minor structural differences between compounds. Although the nTMT threshold was 100-fold higher in ΔD mice compared to WT mice, COCRs of the two strains did not significantly differ at 10^−21^ w/w (*P* = 0.083, Supplementary Information Table ST4). We conclude that WT and ΔD mice have almost identical sensitivity to nTMT (Fig. 2C). The impairment of innate responses of ΔD mice to TMT (while retaining high sensitivity to TMT)^5^ suggests that nTMT supersensitivity is likely to be mediated by sensitive ORs not linked to circuits that process innate chemical signals. This also appears to be the case for carvones and wine lactones.

### Enantiomer odour discrimination paradox in ΔD mice

Next we evaluated the ability of mice to discriminate odours of (−)- vs. (+)-enantiomers. In these experiments, IO post-assays were omitted to prevent animals from being confused by a long test sequence of indiscriminable odour pairs. We found that WT mice were capable of supersensitive discrimination (10^−19^ w/w) of wine lactone enantiomers (larger asterisk in Fig. 1C, Table 1), consistent with their high detection sensitivity. Surprisingly, ΔD mice displayed a >10^14^-fold reduction in discrimination sensitivity for wine lactone enantiomers (threshold of >10^−3^ w/w). They could detect one or both of the (−)- and (+)-wine lactones yet were unable to discriminate them over a wide range of odourant concentrations (10^−3^ to 10^−13^ w/w, blue arrows in Fig. 2A). This mismatch gives rise to an enantiomer odour discrimination paradox. The large difference between detection and discrimination thresholds implies significant overlap in sensitive OR representations of the enantiomers.

**Figure 3:**
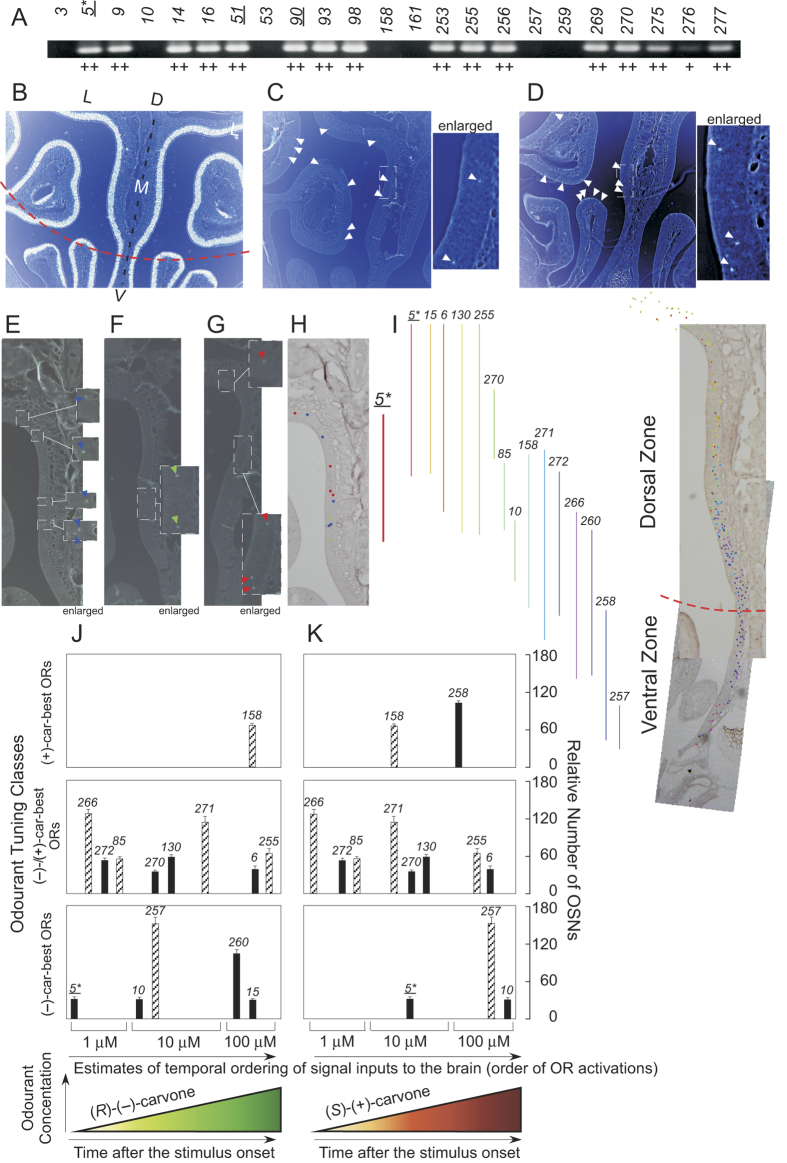
Zonal distribution of carvone ORs. (A) Expression of the zonal marker *O-MACS* in carvone-responsive OSNs. Target-size cDNA products were obtained from 16/103 OSNs. The sequence of cDNA products from 3 OSNs (underlined numbers) were confirmed. Of the 4 OSNs that were specifically and most sensitive to (*R*)-(−)-carvone, one [5*, expressing murine OR *car-c5* (*mORcar-c5*)] was *O-MACS*-positive. It is likely that the deletion of this most sensitive dorsal OR and cognate ORs increases the odour discrimination threshold of ΔD mice for carvone enantiomers. (**B**) Expression of olfactory marker protein in OSNs. Coronal section of the medial part of the mouse olfactory epithelium were *in situ* hybridised with a DIG-labeled antisense RNA probe (negative photography). D, dorsal; V, ventral; M, medial; L, lateral. A border line between dorsal and ventral zones is indicated by the broken red line. (**C**) Expression of *mORcar-c5*. Arrowheads indicate positive OSNs. Enlarged region is indicated by a broken lined box in the low-magnification photograph. (**D**) Expression of *mORcar-b158*. (**E**–**G**), Expression of *mORcar-c5* in three different mice (negative photography). Enlarged region is indicated by a broken lined box in the low-magnification photograph. Positive OSNs are indicated by arrowheads in the enlarged regions and blue, green and red spots in (**H**) respectively. H, Expression region of *mORcar-c5* (red line). (**I**) Expression regions of 15 carvone ORs. Different colours correspond to different ORs. (**J**) Temporally ordered signal inputs to the brain and relative number of the OSNs expressing the (*R*)-(−)-carvone-activated OR. The input orders are based on the OR sensitivities and relative response amplitudes. The numbers of OSNs shown by the hatched bars may be overestimated by potential cross-reactions with other ORs having >85% sequence homology. (**K**) Temporally ordered signal inputs to the brain and relative number of the OSNs expressing the (*S*)-(+)-carvone-activated OR. *Numbers* represent the ORs (Supplementary Information Table ST3).

Similar to wine lactones, the capability of ΔD mice to discriminate carvone enantiomers was also paradoxical, with a 10^10^-fold reduction in discrimination sensitivity (10^−9^ w/w, larger asterisk in Fig. 1F, Table 1). They could detect one or both of the (*R*)-(−)- and (*S*)-(+)-carvones, but were unable to discriminate them over a wide concentration range (10^−11^–10^−17^ w/w, blue arrows in Fig. 2A). Again, this contrasted with relatively consistent detection and discrimination thresholds in WT mice. Our findings indicated that the most sensitive dorsal ORs play critical roles in olfactory signal processing and odour discrimination. Using single-cell PCR, we detected the dorsal zone marker in at least 1 of the 4 most sensitive OSNs for (*R*)-(−)-carvone (*mORcar-c5*, Fig. 3, Supplementary Information Table ST3). The dorsal OR of this OSN is likely to be one of the receptors responsible for supersensitive discrimination of enantiomers by WT mice at threshold. Our results are consistent with a previous report on the capability of ΔD mice to discriminate carvone enantiomers at a high concentration of 6.5 × 10^−2^ w/w, after correcting for total volume of the odourant solution^5^.

The dramatic disruption of enantiomer discrimination caused by ablation of all dorsal ORs in ΔD mice (while retaining high sensitivity to (−)-enantiomers), indicates that the stereoselectivity underlying low threshold discrimination must reside in a small subset of the most sensitive dorsal receptors that contribute disproportionately to the odour representation. Since OR codes for odours, and corresponding glomerular response maps in the bulb, are sparse at threshold, just one or a few highly sensitive, chiral-specific dorsal receptors may be responsible for a large difference between the odour maps of (−)- and (+)-carvone enantiomers. This difference may be sufficient for discrimination of the enantiomers via the conventional olfactory coding scheme based on simple combinatorial representation of different odours by different subsets of responsive receptors^8^. However, we observed significant overlaps of OR codes of carvone enantiomers at higher concentrations, and wine lactone enantiomers at lower concentrations (Supplementary Information Tables ST2, ST3). Such large overlaps, also exhibited by other structurally similar odorant pairs, would make odour discrimination more difficult under the conventional scheme. It has been theorised that decorrelation of strongly overlapped input patterns is an important function of olfactory bulb postsynaptic circuitry^26^,^27^,^28^,^29^. These computations redistribute information across a broad ensemble of bulb output neurons, a process that may take many hundreds of ms^30^. We suggest that the olfactory system may achieve more rapid odour discrimination by selectively filtering presynaptic OR inputs to enhance differences between overlapping patterns. To implement this, we devised a hierarchical odour coding scheme that selectively ranks dorsal ORs with highest sensitivities^10^,^15^. This establishes a weighted combinatorial code emphasising unique sensory qualities (elemental odours) conveyed by the most sensitive dorsal receptors. Our model provides a more nuanced resolution of the enantiomer odour discrimination paradox for both carvone and wine lactone (see below).

## Discussion

### A model explaining the enantiomer odour discrimination paradox in ΔD mice

We previously proposed a hierarchical model of odour coding based on ranking of olfactory receptor sensitivities, that naturally explains stability of odour quality perception for dose-dependently recruiting receptors over a wide concentration range^10^,^15^,^31^. The basic concept is that the earliest arriving signals from the most sensitive, short latency, cognate receptors will be the first to activate inhibitory feedforward pathways in the olfactory cortex through short-latency olfactory bulb tufted cells. These early inhibitory signals from the ventro-rostral part of the anterior piriform cortex (APC_vr_)^14^ receiving signals from a minority of the most sensitive receptors will trigger synchrony of cognate receptor signal inputs to pyramidal cells that selectively evoke ‘elemental’ odour percepts through engaging associative neural pathways. The processing cascade may also act to suppress other odours corresponding to less sensitive, long latency, non-cognate ORs. The model hypothesises that unique elemental odours correspond to a relatively small set of narrowly tuned ORs with highest sensitivities to a target ligand, whereas common elemental odours correspond to more broadly tuned ORs with overlapped sensitivity to multiple ligands. Primary qualities of odour percepts are determined by the unique elemental odours, and are modulated by secondary qualities from the common odours. As we argue in detail below, this model can resolve the enantiomer odour discrimination paradox for both carvones and wine lactones.

### Carvone coding and discrimination

We previously reported that (*R*)-(−)- and (*S*)-(+)-carvones are represented peripherally by different classes of most-sensitive, best-tuned murine ORs: i.e., (*R*)-(−)-carvone-best ORs for the odour of (*R*)-(−)-carvone vs. a combination of (*S*)-(+)-carvone-best ORs and (*R*)-(−)-/(*S*)-(+)-carvone-best (overlapping, equally sensitive) ORs for the odour of (*S*)-(+)-carvone (Supplementary Information Table ST3)^10^. According to our model, the difference in populations of the most sensitive receptors would translate into different perceived elemental odours, enabling WT mice to discriminate between (*R*)-(−)- and (*S*)-(+)-carvones even at the very low detection threshold concentration of 10^−19^ w/w (Fig. 2A). We illustrate specific enantiomer-dependent temporal orders (latencies) of receptor input to the olfactory pathway as predicted by our model, for 15 identified carvone-responsive ORs (Fig. 3J,K). Temporal ordering of activation was inferred from relative response amplitudes of ORs in a heterologous functional expression system^32^ or isolated OSNs^10^ (Supplementary Information Table ST3). For example, *mORcar-n270* and *mORscar-n266* demonstrated slightly greater response amplitudes to (*R*)-(−)- and (*S*)-(+)-carvones, respectively, in a heterologous functional expression system^32^. These sensitivity differences were depicted by slight left shifts of the positions of their signal bars, compared to those of other enantiomers, in Fig. 3J,K. We note, however, that these estimates of temporal ordering are based on indirect measurements (relative response amplitudes), not actual latency times of the responses. Therefore, this prediction of signal ordering is approximate, and may be modified by other determinants of input latency^33^,^34^.

In ΔD mice, the 10^8^-fold and 10^4^-fold reductions in discrimination sensitivity, relative to detection sensitivities for (*R*)-(−)- and (*S*)-(+)-carvones, respectively, can also be explained by our model. We propose that after dorsal OR ablation, the remaining largest tuning class of sensitive ORs comprise (*R*)-(−)-/(*S*)-(+)-carvone-best (overlapping, equally sensitive) receptors. Since ΔD mice have the same detection sensitivity for (*R*)-(−)-carvone as WT mice to (*S*)-(+)-carvone, we suggest that ΔD mice have lost all of the most sensitive (*R*)-(−)-carvone-best ORs expressed in the dorsal zone (e.g. *mORcar-c5*, etc.) but have kept one or two most sensitive ORs in the ventral zone (Supplementary Information Table ST3). Among 15 identified carvone ORs, a deletion of just one most sensitive dorsal OR (*mORcar-c5*) could significantly alter early OR signaling, and change the principal elemental odours of (*R*)-(−)-carvone (Fig. 3J). In this case, the perception of (*R*)-(−)-carvone in ΔD mice may be governed by common elemental odours decoded from signals transduced by the most sensitive (*R*)-(−)-/(*S*)-(+)-carvone-best ORs and adjunctively by (*R*)-(−)-carvone-best ORs in the concentration range of 10^−17^ to 10^−11^ w/w. We previously observed that ORs equally sensitive to (*R*)-(−)- and (*S*)-(+)-carvones became the largest tuning class at concentrations of 10–100 μM^10^ (Supplementary Information Table ST3).

The large reduction in the detection sensitivity to (*S*)-(+)-carvone in ΔD mice may be explained by a loss of nearly all of the most sensitive (*S*)-(+)-carvone-best ORs. The perception of (*S*)-(+)-carvone in ΔD mice may be governed by signals transduced by ORs encoding common elemental odours, possibly accompanied by a weak (*S*)-(+)-carvone unique elemental odour in the upper concentration range of 10^−13^–10^−11^ w/w. In the absence of highly sensitive (*S*)-(+)-carvone-best ORs, only weak signals from common ORs may be relayed to the brain in the lower concentration range (10^−17^–10^−13^ w/w), and these may fall below detection threshold for pyramidal cells of olfactory cortex, due to blocking by initial strong inhibitory signals^14^. Weak subthreshold signals from common ORs may prevent ΔD mice from perceiving any difference between (*R*)-(−)- and (*S*)-(+)-carvones. In our model, the simplest interpretation is that signals of the most sensitive (*R*)-(−)-/(*S*)-(+)-carvone-overlapped equally sensitive ORs dominate the principal elemental odours for both (*R*)-(−)- and (*S*)-(+)-carvones with no emphasis on a weak but unique elemental odour, so that ΔD mice only perceive a common (*R*)-(−)-/(*S*)-(+)-carvone odour and fail to discriminate the enantiomers.

We also note that in our previous study, an NZB mouse and a CBA mouse showed low COCRs close to chance levels at a concentration of 2.5 × 10^−6^ v/v in carvone enantiomer discrimination assays^10^. The purpose of the previous behavioural assay was to assess the enantiomer odour discrimination capability of mice in a high odourant concentration range. Therefore, we focused on odour choice behaviours of mouse in the high concentration range. We also observed unstable scores in a lower concentration range in the previous experimental condition. In order to determine low detection/discrimination thresholds in this study, we modified eight experimental conditions (see Methods). The improved experimental procedure enabled us to measure the extremely low detection and discrimination thresholds (sub-ppq level) of WT mice (Table 1).

### Wine lactone coding and discrimination

ΔD mice maintained a fairly high detection sensitivity for the (−)-wine lactone that was actually comparable to the sensitivity of WT mice to (+)-wine lactone. In contrast, dorsal OR ablation greatly reduced detection sensitivity to (+)-wine lactone. This selective loss of (+)-enantiomer sensitivity was similar to, but more pronounced than the selective loss in carvones. Compared to carvones, WT mice have fewer wine-lactone responsive ORs, and thus wine lactone detection is expected to be more vulnerable to dorsal OR ablation. Indeed, it appears that **Δ**D mice have lost virtually all of the most sensitive wine-lactone ORs, except for (−)-wine-lactone-best ORs. As described in the text, the largest odourant tuning classes of most sensitive murine ORs were comprised of a few (−)-wine-lactone-best ORs and/or a few (−)-/(+)-wine-lactone-best ORs at all tested concentrations (with the exception of (+)-wine lactone at 1 μM) (Supplementary Information Table ST2, Fig. S2). Our data suggest that WT mice may possess no more than two highly sensitive (+)-wine-lactone-best ORs, and that ΔD mice lack all of the most sensitive wine-lactone ORs, except for one or two sensitive (−)-wine-lactone ORs.

According to our model, weak signals from (−)-wine-lactone-best ORs, which are activated by (+)-wine lactone, may be suppressed to fall below threshold levels in the olfactory cortex by initial strong inhibitory signals^14^. Alternatively, loss of the most sensitive target-best ORs may remove strong feedforward inhibition required for integration of signals from cognate ORs, so that the cortical computations that lead to suprathreshold perception of elemental odours would not be triggered in the olfactory cortex. Perceptions of (−)- and (+)-wine lactones in ΔD mice may be governed by elemental odours corresponding to (−)-wine-lactone-best ORs, with the 10^8^-fold less sensitivity to (+)-wine lactone. Lack of significant differences between dominant tuning classes of sensitive ORs and between feedforward inhibitions for emphasising elemental odours would explain the failure of ΔD mice to discriminate the enantiomers.

The precise synaptic and network mechanisms in the olfactory bulb and cortex that could underlie hierarchical odour coding remain to be elucidated. We have suggested that inhibitory pathways from the APC_vr_ through short-latency tufted cells in the olfactory bulb may be essential for similar odour detection and discrimination. In insects, fine discrimination of similar odourants is impaired by desynchronisation of antennal lobe output neurons by picrotoxin, which blocks GABA_A_ receptor-mediated synchrony^35^. In mammals, such inhibitory signal-mediated synchronisation of olfactory bulb mitral/tufted cells could serve to bind signals from selected subsets of cognate (or other) ORs, for downstream readout by coincidence detection in cortical pyramidal cells of the anterior piriform cortex. Recognition, learning and memory of odours is closely tied to enhanced neural synchronisation in the beta band (20 Hz) that is coherent between olfactory bulb and piriform cortex^14^,^36^. In rats, anterior piriform cortical neurons exhibit facilitation and inhibition when stimulated with mixtures of different odourants belonging to distinct perceptual categories, which suggests an enhancement of the category-profile selectivity of individual neurons^37^. Other behavioural assays have also emphasised the importance of early, short-latency OR signals for rapid odour discrimination, as occurs in a single sniff (<200 ms)^38^. In the past, generalised schemes based on relative latencies of parallel receptor inputs have been considered as possible odour codes robust to changing stimulus concentrations^39^. A potential cortical substrate for receptor latency-dependent temporal coding was reported in an optogenetic study showing that piriform cortex neurons are tuned to relative activation latencies of different ORs (and in particular, are order-sensitive)^40^. Optogenetic approaches stand poised to directly test the hierarchical coding hypothesis, especially the roles of dorsal receptors to activate feedforward inhibition through tufted cells projecting to APC_vr_. For example, mice can be trained to discriminate odour representations that differ by photo-activation of a single glomerulus representing input from a single dorsal OR, and furthermore they can detect timing differences in the activation^41^,^42^ and probably detect differences in feedforward inhibition and cortical pyramidal cell activation through short-latency tufted cells and long-latency mitral cells.

## Methods

### Mutant and transgenic mice

Animals were treated in accordance with the Japanese Law (No. 105) and this study was approved by the Institutional Animal Care and Use Committees (RIKEN, University of Tokyo [to which R.K., K.K. and A.T. previously belonged], Osaka Bioscience Institute [to which R.K. and K.K. previously belonged], Kansai Medical University and National Institute of Advanced Industrial Science and Technology). The procedure for mutant mouse generation has been described previously^5^. The *O-MACS* promoter that is expressed only in the olfactory epithelium in a dorsal zone-specific manner was utilised to produce dorsal zone olfactory sensory neuron-defective mutant mice by targeted expression of diphtheria toxin A gene. Mutant mice lacking all olfactory sensory neurons in the dorsal zone were obtained by crossing two knock-in mice: (1) a knock-in mouse in which the coding sequence of *O-MACS* was replaced with that of the *Cre* recombinase gene since the *O-MACS* promoter was native (Cre-O-MACS knock-in mice) and (2) a knock-in mouse in which the *Cre*-inducible diphtheria toxin A gene was introduced into the neuron-specific enolase gene locus (NSE-DTA knock-in mice)^43^.

### Y-maze behavioural assay

We conducted two alternative forced choice behavioural assays in a Y-maze to measure odourant detection thresholds of mice. The Y-maze design was optimised to direct plumes of odourised air along the central axis of each maze arm, maintaining radial concentration gradients between the central axis and arm walls (Supplementary Information Fig. S1). Air through 10 mm inner diameter (I.D.) glass ports centred at the terminal caps was drawn into the maze (0.5 L/min balanced influx, arm I.D. 80 mm, length, 45 cm) by weak negative pressure, and odourised by placing cotton balls moistened with 0.3-mL odourant solution or solvent at the centres of the arm terminal caps. Compared to our previous study with a Y-maze^10^, we were able to measure much lower detection thresholds. To achieve this, we modified eight experimental conditions as described below. First, the influx flow rate was decreased by 0.2 L/min to slow the concentration drop in the odourant source solutions. Air flowed towards the trunk (I.D. 80 mm) of the Y-maze (1.0 L/min efflux). We calculated that a flow rate of 10.6 cm/s in each arm was required for air influx to traverse the 10 mm ports (0.785 cm^2^ cross section)^10^. At this flow rate, transit time for odourised air along each 45 cm maze arm was 4.72 s if the plumes did not expand inside the arms. Because limited plume expansion might slow transit, we waited 10–15 s after terminal caps were attached to the arms. The terminal cap of the trunk was then detached, a mouse placed into it, and immediately reattached, allowing the mouse to run the Y-maze. Air flow from the trunk was maintained continuously, except when the terminal cap was detached to add or remove a mouse.

As a second modified condition, in order to sustain the odourant concentration of flowing air, odourant solution was added every 40 min and 20 min for concentrations higher than 10^−11^ w/w and equal or less than 10^−11^ w/w, respectively. The third condition was that initial stays at the starting port longer than 70 s were treated as incorrect odour choices. In order to remove previously presented odours, air efflux was allowed to continue for 60 s after arm terminal caps with cotton balls were removed. The terminal caps with cotton balls and a small glass funnel for water reward, were independently and randomly exchanged between the two arms. This ensured that mice evenly selected one of the two arms when identical odours were presented in both arms. We often observed a typical behaviour in which mice at the maze junction hesitated and sniffed weak or difficult odours alternately from the two arms for a few seconds.

A fourth modification was that 3-week old mice were used for the initial training instead of 5-week old mice. Each mouse was trained to choose a target odour in an arm at the junction of the maze and run to the end of the arm and drink a drop of water (correct odour choice) (Supplementary Video S1). As the fifth condition change, the upper outside of the maze junction was covered with a piece of cellulose sheet (BEMCOT, M-3, Asahi KASEI, Osaka, Japan, ca. 250-mm length). This modification likely made the mice concentrate on odour choice by preventing them from seeing their trainer’s reactions to their own arm choices. To examine the threshold concentration for odour detection, an odourant solution was serially diluted 100-fold, and the rewarded targets were set to be the odour of the odourant solution vs. that of the solvent (detection assay) and the odour of the (−)-enantiomer solution vs. that of the (+)-enantiomer solution (discrimination assay). As the sixth condition change, we used the (−)-enantiomer, to which mice were more sensitive than the (+)-enantiomer, as the target odourant. This might enable easier odour identification than the (+)-enantiomer is used. The detection or discrimination threshold was defined as the lowest concentration of odourant solution at which the average COCR for the target odour was significantly higher than chance (>59.43%, *P* < 0.05 for 108 trials; χ^2^-test). In order to confirm the consistency of the choice made after completing the assays at the lowest concentration, mice were checked to determine if they were able to: (1) select the target odourant vs. the solvent, at one of the detected concentrations (post-assays, PA) and (2) select one of two identical odours (IO) by chance. In about half the cases, IO assays were not performed to reduce the risk of using non-olfactory cues for the Y-maze arm choice (detection assays of (*S*)-(+)-carvone or nTMT and enantiomer discrimination assays). As the seventh condition change, after each odour detection/discrimination assay series (5–8 weeks per odour) was completed, mice were retrained with 10^−5^–10^−9^-w/w (*R*)-(−)-carvone vs. solvent or (*S*)-(+)-carvone for 2 d to 2 weeks. This likely made mice confirm the odour choice rule.

In this study, 9 WT male mice (C57BL/6 Cr Slc) and 7 ΔD male mice (mixed background of C57BL/6 and 129Svj) were used to select 6 mice of each strain that behaved actively with a trainer. The animals were deprived of water for 1 day prior to the behavioural assays and were provided 1–3 mL water or given free access to water for 30–60 s daily after the assays. Each mouse explored each odour at the same odourant concentration in a set of 18–24 successive trials each day for 2 days. During the 3–4 months of initial odour-choice training, 3 WT mice and 1 ΔD mouse frequently showed initial long-lasting hesitations to run from the starting port or difficulty in learning odour choice rules. We excluded these animals from our analyses. Finally, as the eighth condition change, the number of trials for each mouse was decreased from 24 to 18 trials per day to reduce the risk of using non-olfactory cues for Y-maze arm choice in the late part of trials. The COCR for the last set of each odour at a given concentration was compared with the average values for the initial 18 trials × 6 mice in the same strain (Supplementary Information Table ST1). Statistical analyses of differences between COCRs of WT and ΔD mice were performed with one-way analysis of variance (ANOVA; Supplementary Information Table ST4).

### Odourants

Enantiomer pairs of wine lactones, (3*S*,3a*S*,7a*R*)-3a,4,5,7a-tetrahydro-3,6- dimethylbenzofuran-2(*3H*)-one [(−)-wine lactone] and its (3*R*,3a*R*,7a*S*)-form [(+)-wine lactone], were synthesised according to a previous protocol^44^. The purities of the (−)- and (+)-wine lactones were >99.9% and 100%, respectively, with respect to the angle of optical rotation. The results of the behavioural assays indicated that the (+)-wine lactone sample was either uncontaminated or might have been contaminated by traces (<10^−6^) of the (−)-wine lactone. A carvone enantiomer pair, (*R*)-(−)-carvone and (*S*)-(+)-carvone (>99% purity), and 2,4,5-trimethylthiazole (nTMT, Sigma-Aldrich Co., LLC, St. Louis, MO, USA) were also used.

The odourant solution was diluted with the solvent di(propylene) glycol (dpg). The accuracy of the 100-fold dilution series was examined by the dose-dependency of the peak areas of diluted (*R*)-(−)-carvone solution as a standard using a gas chromatography-mass spectrometry (GC-MS: GC/MS-QP, Shimadzu Corp, Kyoto, Japan) with the BC-W capillary column (50 m, 0.25 mm I.D., 0.15 mm-coated, Shimadzu Corp.). GC heating rate of 10 °C/min (100 °C to 220 °C) and helium carrier gas were used. In order to reduce the viscosity of the odourant solutions in GC-MS measurement, a 1:1 mixture of dpg and chloromethane was used as a solvent. The peak area per unit dose seemed too large for the 10^−9^-w/w odourant solution compared with those at higher concentrations (Supplementary Information Fig. S3, Table ST5). This was likely due to background noise. Thus, the peak areas at the concentrations (C_x_) were corrected by the term -(peak_area_at_10^−9^) + (peak_area_at_10^−3^ × C_x_/10^−3^) (the theoretical curve in Supplementary Information Fig. S3). This result suggests that the detection limit of this GC-MS measurement is approximately 10 ppb for an odourant. The dilution factors were obtained with the ratios of the peak areas per unit dose between subsequent concentrations. The estimated odourant concentrations increased by a factor of less than 10 (Supplementary Information Table ST6). The expected maximum increase in the odourant concentration of 10^−21^ w/w was by a factor of 6.8, with which about 10 target odour molecules are contained in the odourant solution of 0.3 mL in the Y-maze.

Concentrations of odorants for detection and discrimination by the olfactory habituation-dishabituation test used in the previous study^5^ were corrected to those in our diluted solution condition as follows. The odourant solution volumes for the habituation-dishabituation test and the 20-μL solutions for the odour discrimination test were adjusted to 0.3-mL solutions diluted by dpg (M.W. 134.173 Da, density (d.) = 1.02 g/mL). For example, TMT (M.W. 129.22 Da, d. = 1.106 g/mL) of 5.7 × 10^−4^ in the habituation-dishabituation test was equivalent to 1.3 × 10^−8^ w/w [=(3.1 × 10^−11^ M × 129.22 g/M) ÷ (0.3 mL × 1.02 g/mL + 3.1 × 10^−11^ M × 129.22 g/M)] in our experimental condition. For (*S*)-(+)-carvone (M.W. 150.22 Da, d. = 0.96 g/mL), the odourant amount of 20 μL of a 6.2 M solution in the previous odour discrimination test was equivalent to that of 0.3 mL of a 1.9 × 10^−2^ w/w solution [=(20 × 10^−6^ L × 6.2 M/L × 150.22 g/M) ÷ (0.3 mL × 1.02 g/mL − 20 × 10^−6^ L × 6.2 M/L × 150.22 g/M ÷ 0.96 g/mL × 1.02 g/mL + 20 × 10^−6^ L × 6.2 M/L × 150.22 g/M)]. Other parameters were (*R*)-(−)-carvone (M.W. 150.22 Da, d. = 0.959 g/mL) and 2-methylbutyric acid (M.W. 102.132 Da, d. = 0.94 g/mL).

### Ca^2+^ imaging

A total of 53 male mice (BALB/c Cr Slc) were used to isolate 1,746 olfactory sensory neurons. Olfactory epithelium was sampled from the centro-dorsal and postero-ventral portions of the olfactory septum to obtain cells from dorsal and ventral zones, respectively^10^. Sets of time sequenced images of intracellular fura-2 fluorescence were recorded using AQUACOSMOS/RATIO (Hamamatsu Photonics, K.K., Japan). The animals were anesthetised by isoflurane (1–2% gas) and pentobarbital (50 μg/g IP) and sacrificed. Other procedures were as reported previously^10^,^15^. Olfactory sensory neurons were stimulated with Ringer solution containing a target odourant for 4 s.

### *In situ* hybridization

A partial coding sequence of the olfactory marker protein (OMP) gene was cloned as the positive control by single-cell reverse transcription-polymerase chain reaction (RT-PCR)^10^. Different parts of TM2–TM7 regions of 15 carvone OR genes were used to make probes (accession#: DDBJ *LC034567–034581*). Coronal sections of the olfactory epithelia (OE) of 3-week-old male mice were hybridised with a DIG-labeled antisense RNA probe. The probes were prepared by a DIG RNA Labeling kit (Hoffmann-La Roche). Mice were anesthetised with Nembutal (0.1 mL/animal) and perfused intracardially with 4% paraformaldehyde (PFA) in PBS. The nasal cavities were dissected and fixed overnight with 4% PFA in PBS. Then they were decalcified in 0.5 M EDTA overnight at 4 °C, placed in 30% sucrose overnight at 4 °C, and embedded rapidly in O.C.T. compound (Tissue-Tek, Torrance, CA, USA) in liquid nitrogen. Serial coronal sections of the OE (10 μm each) were cut with a JUNG cryostat (Leica, Nussloch, Germany) and collected on 3-aminopropyl-triethoxysilane-coated slide glasses. *In situ* hybridisation were performed as described^45^. After washing, the samples were reacted with alkaline phosphatase-conjugated anti-DIG antibody (Hoffmann-La Roche) (1:1000) for 30 min. Positive cells were stained purple with nitroblue tetrazolium salt and 5-bromo-4-chloro-3-indolyl phosphate toludinium salt. The sections were analysed and photographed on an Axio Plan microscope (Zeiss, Germany). The OE sections were divided into anterior, medial and posterior parts from their morphology. The positive cell numbers were counted on the selected sections from all the three parts equally. Potential cross-reactions with other ORs having >85% sequence homology were based on previously reported observations^46^.

### Polymerase chain reaction (PCR)

A cDNA fragment specific to the zonal marker *O-MACS* was amplified by single-cell PCR using 10-fold diluted single-cell cDNA that had been prepared and stored at −20 °C in our previous study^10^. The primers 5′-CACTgATAgAgCACCCAgCA-3′ and 5′-TATgCTCTTCCCCATTCCTg-3′ were designed from an intron-containing region close to the C terminus of the *O-MACS* gene. PCR was performed using HotStarTaq DNA polymerase (QIAGEN, Inc.) using the following schedule: 95 °C for 15 min followed by 25 cycles of 95 °C for 30 s, 60 °C for 30 s, and 72 °C for 30 s. The PCR product, as expected, was 274-bp long.

## Additional Information

**How to cite this article**: Sato, T. *et al.* Supersensitive detection and discrimination of enantiomers by dorsal olfactory receptors: evidence for hierarchical odour coding. *Sci. Rep.* **5**, 14073; doi: 10.1038/srep14073 (2015).

## Supplementary Material

Supplementary Information

Supplementary Video 1

## Figures and Tables

**Table 1:** Detection/discrimination threshold concentrations for two pairs of enantiomers.

**odourants**	**mouse strains**	**detection thresholds of odourant solutions**		**ratio of detection thresholds ((+)- to (−)-enantiomer)**		**discrimination thresholds between enantiomers**	
		**(w/w)**	**rel.** Δ**D sen.**	**mouse**	**human**	**(w/w)**	**rel.** Δ**D sen.**
(−)-wnl	wild type	1.0 × 10^−21^	1.0 × 10^−2^	1.0 × 10^2^	ca. 10^8 ref#4^	1.0 × 10^−17^	<10^−14^
	ΔD	1.0 × 10^−19^		1.0 × 10^8^		>10^−3^	
(+)-wnl	wild type	1.0 × 10^−19^	1.0 × 10^−8^	—	—	—	—
	ΔD	1.0 × 10^−11^		—		—	
(−)-car	wild type	1.0 × 10^−19^	1.0 × 10^−2^	1.0 × 10^2^	8.6 × 10^0 ref#3^	1.0 × 10^−19^	1.0 × 10^−10^
	ΔD	1.0 × 10^−17^		1.0 × 10^4^		1.0 × 10^−9^	
(+)-car	wild type	1.0 × 10^−17^	1.0 × 10^−4^	—	—	—	—
	ΔD	1.0 × 10^−13^		—		—	
nTMT	wild type	1.0 × 10^−21^	1.0 × 10^−2^	—	—	—	—
	ΔD	1.0 × 10^−19^		—		—	

The detection and discrimination thresholds were obtained as the lowest odourant concentrations with the average values of the correct odour choice rates (COCR) for 6 mice of the same strain (18 trials × 6 mice) greater than 59.43% (*P* = 0.05 for 108 trials). The threshold differences are shown as the ratios of wild-type to ΔD mice (rel. ΔD sen.). The odourants were diluted in di(propylene glycol) (w/w) in a 100-fold dilution series. Odourant abbreviations: (−)-wnl, (3*S*,3a*S*,7a*R*)-wine lactone; (+)-wnl, (3*R*,3a*R*,7a*S*)-wine lactone; (−)-car, (*R*)-(−)-carvone; (+)-car, (*S*)-(+)-carvone; nTMT, non-dihydrogenated TMT.
