# Supplementary material for: Supersensitive detection and discrimination of enantiomers by dorsal olfactory receptors: evidence for hierarchical odour coding
Source: Sci Rep. 2015 Sep 11;5:14073. doi: 10.1038/srep14073 (PMC4566093; doi:10.1038/srep14073)
Supplement: Supplementary Information [file srep14073-s1.pdf]

# Supplementary Information
# Supersensitive detection and discrimination of enantiomers by dorsal olfactory receptors: evidence for hierarchical odour coding

Takaaki Sato[1,*], Reiko Kobayakawa[2], Ko Kobayakawa[2], Makoto Emura[3], Shigeyoshi Itohara[4], Miwako Kizumi[1], Hiroshi Hamana[1], Akio Tsuboi[5] and Junzo Hirono[1]

[1] Biomedical Research Institute, National Institute of Advanced Industrial Science and Technology, Hyogo 661-0974, Japan. [2] Institute of Biomedical Science, Kansai Medical University, Osaka 573-1010, Japan. [3] Takasago International Corporation, Kanagawa 254-0073, Japan. [4] Laboratory for Behavioral Genetics, Brain Science Institute, RIKEN, Saitama 351-0198, Japan. [5] Research Institute of Frontier Medicine, Nara Medical University, Nara 634-8521, Japan.

Other Supplementary Information for this manuscript includes the following:
(available at www.nature.com/scientificreports/)

Video S1. Odour setting and correct odour choice behaviour in the Y-maze.

**Figure S1.** A schematic diagram of the Y-maze. Two alternative forced choice assays, with mice choosing between odourant and solvent solutions, or between pairs of enantiomeric odourant solutions, were conducted in a Y-maze. Cotton balls moistened with diluted odourants were placed at the inlet ports of the maze arms. Odour vapour flows were guided into the Y-maze at the centres of the cavities by weak negative pressure. Flow rate at inlet port was adjusted to 0.5 L/min by regulating leakage through a 3-way valve. Runs to the target odour source (correct odour choice) against the (+)-enantiomers or the solvent were rewarded with a drop of water. Rewarded side was randomly switched by independently exchanging the terminal caps and funnels between the arms. A mouse was picked up in the start cup and set in the Y-maze to initiate a trial running to the end of one arm. When the mouse chose the wrong side, both terminal caps were removed to prevent the mouse from drinking water from the funnel. Each mouse ran and chose one of the two odours 18 to 24 times per day. The sums of the initial 18 trials for 6 mice of the same strain were statistically analysed.

**Figure S2.** Odour responses of the most sensitive OSNs for wine lactones in mice. Odour responses were measured using intracellular $Ca^{2+}$-imaging. A, OSN tuned to 10 nM (–)-wnl. B, OSN tuned to 10 nM (–)-wnl and (+)-wnl. C, OSN tuned to 1 μM (+)-wnl. D and E, OSNs tuned to 1 μM (–)-wnl. (–)-ro: (2*S*,4*R*)-(–)-cis-rose oxide; (+)-ro: (2*R*,4*S*)-(+)-cis-rose oxide. The arrows indicate the time at which odourant solutions of the indicated concentrations were applied to the OSNs for 4 s. The odourants that evoked characteristic odour responses are indicated by the asterisks. All OSNs, except for that shown in E, were obtained from the dorsal zone.

**Figure S3.** Dose-dependency of the peak areas of diluted odourant solutions in gas chromatography-mass spectrometry (GC-MS). The peak area per unit dose seemed too large for the $10^{-9}$-w/w odourant solution compared with those at higher concentrations. This was likely due to background noise. This result suggests that the detection limit of this GC-MS measurement is approximately 10 ppb for (*R*)-(–)-carvone. The theoretical curve (dashed line) is the plot of the peak areas at the concentrations ($C_x$) with the correction by the term -(peak_area_at_$10^{-9}$) + (peak_area_at_$10^{-3}$ × $C_x/10^{-3}$).

**Table ST1.** COCRs of WT and ΔD mice in a Y-maze for serial 100-fold diluted odourants.

**Table ST2.** Dose-dependent subpopulations of wine lactone-responsive OSNs/ORs. Of 1,746 olfactory sensory neurons (OSNs) (1,207 dorsal zone: 539 ventral zone) assayed by

$Ca^{2+}$ imaging, 2, 5, 17 and 46 OSNs/ORs responded to either or both wine lactone enantiomers at 10-100 nM, 1, 10 and 10 μM, respectively. They were classified by their tuning to target enantiomers of wine lactones. Target-best OSNs/ORs* are those more sensitive to the target enantiomer than to the non-target enantiomer. Percentage of each class responsive to the target at a given concentration is listed in the lower row. OR overlap indicates percentage (numbers) of overlapping OSN/OR in enantiomer OR codes at each concentration. (–)-wnl, (–)-wine lactone; (+)-wnl, (+)-wine lactone.

**Table ST3.** Dose-dependent subpopulations of carvone-responsive OSNs/ORs. Of 2,740 OSNs/ORs assayed by $Ca^{2+}$ imaging, 13 and 89 OSNs/ORs (dorsal zone: ventral zone) responded to either or both carvone enantiomers at 1 and 10 μM, respectively, and were classified by their tuning to target odourants. Target-best OSNs/ORs* are those more sensitive to the target enantiomer than to the non-target enantiomer. Percentage of each class responsive to the target at a given concentration is listed in the lower row. The percentage† indicates OSNs/ORs with response amplitudes less than the half of the others. The percentages in parentheses indicate OSNs/ORs that were prepared from uncertain zones and counted in each zone by a factor of 1/2. OR overlap indicates percentage (numbers) of overlapping OSN/OR in enantiomer OR codes at each concentration. The largest class‡ is the sum of (+)-car and (+)-car/others OSNs/ORs. In the lower part, relative response amplitudes of 15 identified carvone ORs are shown with the lowest responsive concentrations (μM) for target (left column) and non-target carvone in parentheses (Fig. 3).

**Table ST4.** Significance of the COCR differences between WT and ΔD mice.

**Table ST5.** Peak areas of GC-MS and calculation of the dilution factors for the 100-fold dilution series. The dilution factors were obtained as the ratios of peak areas per unit dose between subsequent concentrations. (*R*)-(−)-carvone was used as the standard odourant. The dose-dependency of the peak areas was shown in Supplementary Information Fig. S3.

**Table ST6.** Estimated odourant concentrations. Averaged dilution factor, $D_{avg} = 1.0109 \pm 0.0515$, and the maximum dilution factor, $D_{max} = 1.21$, are shown in Supplementary Information Table ST5.

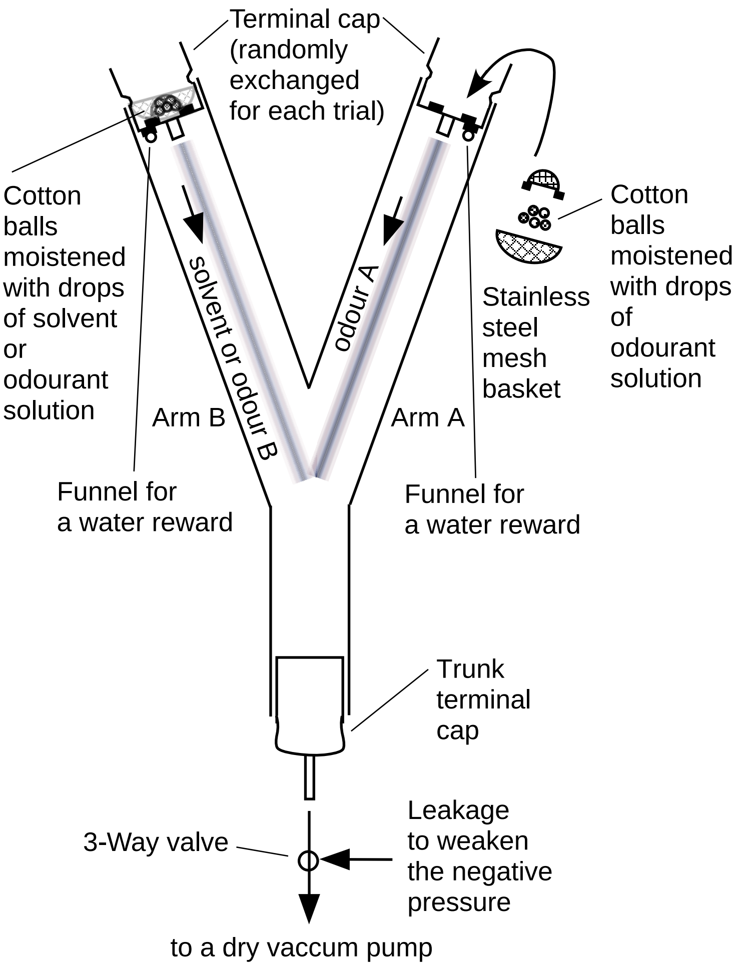


**Figure S1.** A schematic diagram of the Y-maze. Two alternative forced choice assays, with mice choosing between odourant and solvent solutions, or between pairs of enantiomeric odourant solutions, were conducted in a Y-maze. Cotton balls moistened with diluted odourants were placed at the inlet ports of the maze arms. Odour vapour flows were guided into the Y-maze at the centres of the cavities by weak negative pressure. Flow rate at inlet port was adjusted to 0.5 L/min by regulating leakage through a 3-way valve. Runs to the target odour source (correct odour choice) vs. the (+)-enantiomers or the solvent were rewarded with a drop of water. Rewarded side was randomly switched by independently exchanging the terminal caps and funnels between the arms. A mouse was picked up in the trunk terminal cap and set in the Y-maze to initiate a trial running to the end of one arm. When the mouse chose the wrong side, both terminal caps were removed to prevent the mouse from drinking water from the funnel. Each mouse ran and chose one of the two odours 18 to 24 times per day. Sums of initial 18 trials for 6 mice were statistically analysed.

| Odourant pairs | COCRs of individual WT mice (%) | | | | | | avg. of 3 mice (wt1-3) | S.E.M. | avg. of 3 mice (wt4-6) | S.E.M. | avg. of 6 WT mice | S.E.M. | COCR for $P$ = 0.05 |
|---|---|---|---|---|---|---|---|---|---|---|---|---|---|
| | wt1 | wt2 | wt3 | wt4 | wt5 | wt6 | | | | | | | |
| (-)-wnl vs. solvent | | | | | | | | | | | | | |
| $10^{-3}$ (-)-wnl vs. solvent | 100.0 | 72.2 | 88.9 | 77.8 | 83.3 | 100.0 | 87.0 | 8.1 | 87.0 | 6.7 | 87.0 | 4.7 | 59.4 |
| $10^{-5}$ (-)-wnl vs. solvent | 77.8 | 77.8 | 83.3 | 77.8 | 77.8 | 72.2 | 79.6 | 1.9 | 75.9 | 1.9 | 77.8 | 1.4 | 59.4 |
| $10^{-7}$ (-)-wnl vs. solvent | 88.9 | 66.7 | 66.7 | 83.3 | 66.7 | 61.1 | 74.1 | 7.4 | 70.4 | 6.7 | 72.2 | 4.5 | 59.4 |
| $10^{-9}$ (-)-wnl vs. solvent | 77.8 | 72.2 | 61.1 | 83.3 | 72.2 | 77.8 | 70.4 | 4.9 | 77.8 | 3.2 | 74.1 | 3.1 | 59.4 |
| $10^{-11}$ (-)-wnl vs. solvent | 72.2 | 66.7 | 72.2 | 88.9 | 88.9 | 88.9 | 70.4 | 1.9 | 88.9 | 0.0 | 79.6 | 4.2 | 59.4 |
| $10^{-13}$ (-)-wnl vs. solvent | 72.2 | 66.7 | 72.2 | 94.4 | 83.3 | 88.9 | 70.4 | 1.9 | 88.9 | 3.2 | 79.6 | 4.5 | 59.4 |
| $10^{-15}$ (-)-wnl vs. solvent | 83.3 | 77.8 | 72.2 | 77.8 | 83.3 | 83.3 | 77.8 | 3.2 | 81.5 | 1.9 | 79.6 | 1.9 | 59.4 |
| $10^{-17}$ (-)-wnl vs. solvent | 66.7 | 72.2 | 72.2 | 83.3 | 83.3 | 77.8 | 70.4 | 1.9 | 81.5 | 1.9 | 75.9 | 2.7 | 59.4 |
| $10^{-19}$ (-)-wnl vs. solvent | 77.8 | 61.1 | 61.1 | 77.8 | 72.2 | 77.8 | 66.7 | 5.6 | 75.9 | 1.9 | 71.3 | 3.3 | 59.4 |
| $10^{-21}$ (-)-wnl vs. solvent | 66.7 | 77.8 | 61.1 | 72.2 | 61.1 | 66.7 | 68.5 | 4.9 | 66.7 | 3.2 | 67.6 | 2.7 | 59.4 |
| $10^{-23}$ (-)-wnl vs. solvent | 61.1 | 61.1 | 44.4 | 66.7 | 61.1 | 61.1 | 55.6 | 5.6 | 63.0 | 1.9 | 59.3 | 3.1 | 59.4 |
| $10^{-25}$ (-)-wnl vs. solvent | 55.6 | 44.4 | 55.6 | 50.0 | 44.4 | 66.7 | 51.9 | 3.7 | 53.7 | 6.7 | 52.8 | 3.4 | 59.4 |
| PO assays | | | | | | | | | | | | | |
| $10^{-13}$ (-)-wnl vs. solvent | 66.7 | 55.6 | 83.3 | 72.2 | 72.2 | 83.3 | 68.5 | 8.1 | 75.9 | 3.7 | 72.2 | 4.3 | 59.4 |
| $10^{-13}$ (-)-wnl vs. (-)-wnl | 55.6 | 50.0 | 61.1 | 44.4 | 50.0 | 50.0 | 55.6 | 3.2 | 48.1 | 1.9 | 51.9 | 2.3 | 59.4 |
| (+)-wnl vs. solvent | | | | | | | | | | | | | |
| $10^{-3}$ (+)-wnl vs. solvent | 94.4 | 77.8 | 66.7 | 75.0 | 75.0 | 70.8 | 79.6 | 8.1 | 73.6 | 1.4 | 76.6 | 3.9 | 59.4 |
| $10^{-5}$ (+)-wnl vs. solvent | 66.7 | 66.7 | 77.8 | 83.3 | 83.3 | 83.3 | 72.2 | 5.6 | 83.3 | 0.0 | 77.8 | 3.5 | 59.4 |
| $10^{-7}$ (+)-wnl vs. solvent | 83.3 | 77.8 | 66.7 | 75.0 | 83.3 | 79.2 | 72.2 | 8.5 | 79.2 | 2.4 | 75.7 | 4.2 | 59.4 |
| $10^{-9}$ (+)-wnl vs. solvent | 77.8 | 88.9 | 88.9 | 79.2 | 79.2 | 95.8 | 81.5 | 3.7 | 84.7 | 5.6 | 83.1 | 3.1 | 59.4 |
| $10^{-11}$ (+)-wnl vs. solvent | 72.2 | 77.8 | 55.6 | 79.2 | 79.2 | 83.3 | 68.5 | 6.7 | 80.6 | 1.4 | 74.5 | 4.1 | 59.4 |
| $10^{-13}$ (+)-wnl vs. solvent | 88.9 | 88.9 | 77.8 | 75.0 | 83.3 | 83.3 | 85.2 | 3.7 | 80.6 | 2.8 | 82.9 | 2.3 | 59.4 |
| $10^{-15}$ (+)-wnl vs. solvent | 83.3 | 77.8 | 77.8 | 75.0 | 79.2 | 75.0 | 74.1 | 6.7 | 76.4 | 1.4 | 75.2 | 3.1 | 59.4 |
| $10^{-17}$ (+)-wnl vs. solvent | 83.3 | 77.8 | 77.8 | 75.0 | 62.5 | 75.0 | 79.6 | 1.9 | 70.8 | 4.2 | 75.2 | 2.8 | 59.4 |
| $10^{-19}$ (+)-wnl vs. solvent | 77.8 | 83.3 | 88.9 | 66.7 | 75.0 | 66.7 | 79.6 | 1.9 | 69.4 | 2.8 | 74.5 | 2.7 | 59.4 |
| $10^{-21}$ (+)-wnl vs. solvent | 50.0 | 50.0 | 33.3 | 62.5 | 58.3 | 58.3 | 44.4 | 5.6 | 59.7 | 1.4 | 52.1 | 4.3 | 59.4 |
| $10^{-23}$ (+)-wnl vs. solvent | | | | 58.3 | 50.0 | 58.3 | | | 55.6 | 2.8 | | | 63.3 |
| PO assays | | | | | | | | | | | | | |
| $10^{-19}$ (+)-wnl vs. solvent | 72.2 | 72.2 | 77.8 | 77.8 | 77.8 | 66.7 | 70.4 | 1.9 | 74.1 | 3.7 | 72.2 | 2.0 | 59.4 |
| $10^{-19}$ (+)-wnl vs. (+)-wnl | 50.0 | 38.9 | 55.6 | 33.3 | 33.3 | 66.7 | 46.3 | 3.7 | 44.4 | 11.1 | 45.4 | 5.3 | 59.4 |
| (-)-wnl vs. (+)-wnl | | | | | | | | | | | | | |
| $10^{-3}$ (-)-wnl vs. (+)-wnl | 55.6 | 77.8 | 66.7 | 72.2 | 66.7 | 77.8 | 66.7 | 6.4 | 72.2 | 3.2 | 69.4 | 3.4 | 59.4 |
| $10^{-5}$ (-)-wnl vs. (+)-wnl | 55.6 | 55.6 | 66.7 | 46.7 | 40.0 | 61.1 | 59.3 | 3.7 | 49.3 | 6.2 | 54.3 | 3.9 | 59.4 |
| $10^{-7}$ (-)-wnl vs. (+)-wnl | 83.3 | 88.9 | 77.8 | 55.6 | 55.6 | 77.8 | 83.3 | 3.2 | 63.0 | 7.4 | 73.1 | 5.8 | 59.4 |
| $10^{-9}$ (-)-wnl vs. (+)-wnl | 72.2 | 66.7 | 61.1 | 61.1 | 72.2 | 77.8 | 66.7 | 3.2 | 70.4 | 4.9 | 68.5 | 2.7 | 59.4 |
| $10^{-11}$ (-)-wnl vs. (+)-wnl | 77.8 | 61.1 | 55.6 | 50.0 | 61.1 | 66.7 | 64.8 | 6.7 | 59.3 | 4.9 | 62.0 | 3.9 | 59.4 |
| $10^{-13}$ (-)-wnl vs. (+)-wnl | 88.9 | 88.9 | 66.7 | 61.1 | 61.1 | 66.7 | 81.5 | 7.4 | 63.0 | 1.9 | 72.2 | 5.4 | 59.4 |
| $10^{-15}$ (-)-wnl vs. (+)-wnl | 72.2 | 66.7 | 55.6 | 66.7 | 83.3 | 61.1 | 64.8 | 4.9 | 70.4 | 6.7 | 67.6 | 3.9 | 59.4 |
| $10^{-17}$ (-)-wnl vs. (+)-wnl | 44.4 | 55.6 | 50.0 | 77.8 | 83.3 | 55.6 | 50.0 | 3.2 | 72.2 | 8.5 | 61.1 | 6.4 | 59.4 |
| $10^{-19}$ (-)-wnl vs. (+)-wnl | 50.0 | 61.1 | 55.6 | 66.7 | 61.1 | 50.0 | 55.6 | 3.2 | 59.3 | 4.9 | 57.4 | 2.7 | 59.4 |
| $10^{-21}$ (-)-wnl vs. (+)-wnl | 50.0 | 55.6 | 72.2 | 66.7 | 61.1 | 44.4 | 59.3 | 6.7 | 57.4 | 6.7 | 58.3 | 4.2 | 59.4 |
| $10^{-23}$ (-)-wnl vs. (+)-wnl | 66.7 | 55.6 | 44.4 | 55.6 | 55.6 | 44.4 | 55.6 | 6.4 | 51.9 | 3.7 | 53.7 | 3.4 | 59.4 |
| PO assays | | | | | | | | | | | | | |
| $10^{-13}$ (-)-wnl vs. (+)-wnl | 72.2 | 77.8 | 94.4 | 66.7 | 61.1 | 72.2 | 81.5 | 6.7 | 66.7 | 3.2 | 74.1 | 4.7 | 59.4 |
| $10^{-13}$ (-)-wnl vs. solvent | 83.3 | 83.3 | 66.7 | 88.9 | 94.4 | 88.9 | 79.2 | 4.2 | 90.7 | 1.9 | 84.3 | 3.9 | 59.4 |
| (-)-car vs. solvent | | | | | | | | | | | | | |
| $10^{-3}$ (-)-car vs. solvent | 83.3 | 77.8 | 83.3 | 72.2 | 100.0 | 72.2 | 81.5 | 1.9 | 81.5 | 9.3 | 81.5 | 4.2 | 59.4 |
| $10^{-5}$ (-)-car vs. solvent | 77.8 | 83.3 | 83.3 | 72.2 | 94.4 | 83.3 | 81.5 | 1.9 | 83.3 | 6.4 | 82.4 | 3.0 | 59.4 |
| $10^{-7}$ (-)-car vs. solvent | 75.0 | 75.0 | 79.2 | 66.7 | 61.1 | 77.8 | 76.4 | 1.4 | 68.5 | 4.9 | 72.5 | 2.9 | 59.4 |
| $10^{-9}$ (-)-car vs. solvent | 55.6 | 66.7 | 61.1 | 83.3 | 66.7 | 72.2 | 61.1 | 3.2 | 74.1 | 4.9 | 67.6 | 3.9 | 59.4 |
| $10^{-11}$ (-)-car vs. solvent | 50.0 | 72.2 | 72.2 | 83.3 | 94.4 | 83.3 | 64.8 | 7.4 | 87.0 | 3.7 | 75.9 | 6.2 | 59.4 |
| $10^{-13}$ (-)-car vs. solvent | 61.1 | 61.1 | 72.2 | 83.3 | 88.9 | 88.9 | 64.8 | 3.7 | 87.0 | 1.9 | 75.9 | 5.3 | 59.4 |
| $10^{-15}$ (-)-car vs. solvent | 77.8 | 66.7 | 72.2 | 77.8 | 61.1 | 77.8 | 72.2 | 3.2 | 72.2 | 5.6 | 72.2 | 2.9 | 59.4 |
| $10^{-17}$ (-)-car vs. solvent | 66.7 | 72.2 | 61.1 | 88.9 | 77.8 | 77.8 | 66.7 | 3.2 | 81.5 | 3.7 | 74.1 | 4.0 | 59.4 |
| $10^{-19}$ (-)-car vs. solvent | 72.2 | 72.2 | 72.2 | 66.7 | 61.1 | 61.1 | 72.2 | 0.0 | 63.0 | 1.9 | 67.6 | 2.2 | 59.4 |
| $10^{-21}$ (-)-car vs. solvent | 66.7 | 61.1 | 55.6 | 55.6 | 44.4 | 55.6 | 61.1 | 3.2 | 51.9 | 3.7 | 56.5 | 3.0 | 59.4 |
| $10^{-23}$ (-)-car vs. solvent | 61.1 | 61.1 | 61.1 | 50.0 | 55.6 | 55.6 | 61.1 | 0.0 | 53.7 | 1.9 | 57.4 | 1.9 | 59.4 |
| $10^{-25}$ (-)-car vs. solvent | 55.6 | 61.1 | 55.6 | 61.1 | 38.9 | 44.4 | 57.4 | 1.9 | 48.1 | 6.7 | 52.8 | 3.7 | 59.4 |
| PO assays | | | | | | | | | | | | | |
| $10^{-11}$ (-)-car vs. solvent | 72.2 | 77.8 | 72.2 | 70.8 | 79.2 | 70.8 | 74.1 | 1.9 | 73.6 | 2.8 | 74.1 | 1.9 | 59.4 |
| $10^{-11}$ (-)-car vs. (-)-car | 44.4 | 61.1 | 44.4 | 38.9 | 61.1 | 44.4 | 50.0 | 5.6 | 48.1 | 6.7 | 50.0 | 5.6 | 59.4 |

**Table ST1.** COCRs of WT and ΔD mice in a Y-maze for serial 100-fold diluted odourants.

| Odourant pairs | COCRs of individual WT mice (%) | | | | | | avg. of 3 mice (wt1-3) | S.E.M. | avg. of 3 mice (wt4-6) | S.E.M. | avg. of 6 WT mice | S.E.M. | COCR for $P$ = 0.05 |
|---|---|---|---|---|---|---|---|---|---|---|---|---|---|
| | wt1 | wt2 | wt3 | wt4 | wt5 | wt6 | | | | | | | |
| (+)-car vs. solvent | | | | | | | | | | | | | |
| $10^{-3}$ (+)-car vs. solvent | 72.2 | 72.2 | 72.2 | 94.4 | 83.3 | 83.3 | 72.2 | 0.0 | 87.0 | 3.7 | 79.6 | 3.7 | 59.4 |
| $10^{-5}$ (+)-car vs. solvent | 83.3 | 66.7 | 72.2 | 88.9 | 77.8 | 88.9 | 74.1 | 4.9 | 85.2 | 3.7 | 79.6 | 3.7 | 59.4 |
| $10^{-7}$ (+)-car vs. solvent | 77.8 | 77.8 | 88.9 | 77.8 | 72.2 | 83.3 | 81.5 | 3.7 | 77.8 | 3.2 | 79.6 | 2.3 | 59.4 |
| $10^{-9}$ (+)-car vs. solvent | 72.2 | 61.1 | 66.7 | 66.7 | 61.1 | 61.1 | 66.7 | 3.2 | 63.0 | 1.9 | 64.8 | 1.9 | 59.4 |
| $10^{-11}$ (+)-car vs. solvent | 66.7 | 77.8 | 77.8 | 66.7 | 61.1 | 77.8 | 74.1 | 3.7 | 68.5 | 4.9 | 71.3 | 3.0 | 59.4 |
| $10^{-13}$ (+)-car vs. solvent | 83.3 | 77.8 | 66.7 | 66.7 | 72.2 | 66.7 | 75.9 | 4.9 | 68.5 | 1.9 | 72.2 | 2.9 | 59.4 |
| $10^{-15}$ (+)-car vs. solvent | 72.2 | 77.8 | 88.9 | 61.1 | 66.7 | 66.7 | 79.6 | 4.9 | 64.8 | 1.9 | 72.2 | 4.1 | 59.4 |
| $10^{-17}$ (+)-car vs. solvent | 61.1 | 72.2 | 77.8 | 61.1 | 61.1 | 55.6 | 70.4 | 4.9 | 59.3 | 1.9 | 64.8 | 3.4 | 59.4 |
| $10^{-19}$ (+)-car vs. solvent | 55.6 | 55.6 | 61.1 | 55.6 | 55.6 | 61.1 | 57.4 | 1.9 | 57.4 | 1.9 | 57.4 | 1.2 | 59.4 |
| $10^{-21}$ (+)-car vs. solvent | 61.1 | 61.1 | 50.0 | 61.1 | 66.7 | 61.1 | 57.4 | 3.7 | 63.0 | 1.9 | 60.2 | 2.2 | 59.4 |
| $10^{-23}$ (+)-car vs. solvent | 50.0 | 50.0 | 55.6 | 55.6 | 50.0 | 55.6 | 51.9 | 1.9 | 53.7 | 1.9 | 52.8 | 1.2 | 59.4 |
| PO assays | | | | | | | | | | | | | |
| $10^{-5}$ (-)-car vs. solvent | 66.7 | 66.7 | 77.8 | 83.3 | 83.3 | 77.8 | 70.4 | 3.7 | 81.5 | 1.9 | 75.9 | 3.1 | 59.4 |
| (-)-car vs. (+)-car | | | | | | | | | | | | | |
| $10^{-3}$ (-)-car vs. (+)-car | 72.2 | 94.4 | 72.2 | 77.8 | 83.3 | 66.7 | 79.6 | 7.4 | 75.9 | 4.9 | 77.8 | 4.1 | 59.4 |
| $10^{-5}$ (-)-car vs. (+)-car | 72.2 | 66.7 | 83.3 | 72.2 | 66.7 | 77.8 | 74.1 | 4.9 | 72.2 | 3.2 | 73.1 | 2.7 | 59.4 |
| $10^{-7}$ (-)-car vs. (+)-car | 66.7 | 72.2 | 66.7 | 83.3 | 72.2 | 88.9 | 68.5 | 1.9 | 81.5 | 4.9 | 75.0 | 3.7 | 59.4 |
| $10^{-9}$ (-)-car vs. (+)-car | 61.1 | 61.1 | 72.2 | 66.7 | 61.1 | 72.2 | 64.8 | 3.7 | 66.7 | 3.2 | 65.7 | 2.2 | 59.4 |
| $10^{-11}$ (-)-car vs. (+)-car | 66.7 | 77.8 | 83.3 | 61.1 | 66.7 | 61.1 | 75.9 | 4.9 | 63.0 | 1.9 | 69.4 | 3.7 | 59.4 |
| $10^{-13}$ (-)-car vs. (+)-car | 55.6 | 61.1 | 55.6 | 72.2 | 50.0 | 77.8 | 57.4 | 1.9 | 66.7 | 8.5 | 62.0 | 4.4 | 59.4 |
| $10^{-15}$ (-)-car vs. (+)-car | 66.7 | 66.7 | 66.7 | 66.7 | 55.6 | 88.9 | 66.7 | 0.0 | 70.4 | 9.8 | 68.5 | 4.5 | 59.4 |
| $10^{-17}$ (-)-car vs. (+)-car | 72.2 | 61.1 | 72.2 | 61.1 | 55.6 | 77.8 | 68.5 | 3.7 | 64.8 | 6.7 | 66.7 | 3.5 | 59.4 |
| $10^{-19}$ (-)-car vs. (+)-car | 61.1 | 61.1 | 66.7 | 61.1 | 55.6 | 61.1 | 63.0 | 1.9 | 59.3 | 1.9 | 61.1 | 1.4 | 59.4 |
| $10^{-21}$ (-)-car vs. (+)-car | 44.4 | 50.0 | 61.1 | 55.6 | 50.0 | 61.1 | 51.9 | 4.9 | 55.6 | 3.2 | 53.7 | 2.7 | 59.4 |
| $10^{-23}$ (-)-car vs. (+)-car | 55.6 | 44.4 | 61.1 | 44.4 | 55.6 | 55.6 | 53.7 | 4.9 | 51.9 | 3.7 | 52.8 | 2.8 | 59.4 |
| PO assays | | | | | | | | | | | | | |
| $10^{-5}$ (-)-car vs. solvent | 94.4 | 66.7 | 83.3 | 88.9 | 66.7 | 83.3 | 81.5 | 8.1 | 79.6 | 6.7 | 80.6 | 4.7 | 59.4 |
| nTMT vs dpg | | | | | | | | | | | | | |
| | | | | | | | | | | | | | |
| $10^{-9}$ nTMT vs. solvent | 94.4 | 61.1 | 50.0 | 66.7 | 66.7 | 72.2 | 68.5 | 13.4 | 68.5 | 1.9 | 68.5 | 6.0 | 59.4 |
| $10^{-11}$ nTMT vs. solvent | 66.7 | 66.7 | 55.6 | 66.7 | 72.2 | 77.8 | 63.0 | 3.7 | 72.2 | 3.2 | 67.6 | 3.0 | 59.4 |
| $10^{-13}$ nTMT vs. solvent | 83.3 | 72.2 | 61.1 | 83.3 | 83.3 | 88.9 | 72.2 | 6.4 | 85.2 | 1.9 | 78.7 | 4.2 | 59.4 |
| $10^{-15}$ nTMT vs. solvent | 83.3 | 72.2 | 61.1 | 77.8 | 66.7 | 94.4 | 72.2 | 6.4 | 79.6 | 8.1 | 75.9 | 4.9 | 59.4 |
| $10^{-17}$ nTMT vs. solvent | 77.8 | 77.8 | 66.7 | 61.1 | 55.6 | 72.2 | 74.1 | 3.7 | 63.0 | 4.9 | 68.5 | 3.7 | 59.4 |
| $10^{-19}$ nTMT vs. solvent | 66.7 | 66.7 | 61.1 | 66.7 | 66.7 | 72.2 | 64.8 | 1.9 | 68.5 | 1.9 | 66.7 | 1.4 | 59.4 |
| $10^{-21}$ nTMT vs. solvent | 88.9 | 55.6 | 55.6 | 72.2 | 50.0 | 61.1 | 66.7 | 11.1 | 61.1 | 6.4 | 63.9 | 5.9 | 59.4 |
| $10^{-23}$ nTMT vs. solvent | 66.7 | 61.1 | 55.6 | 55.6 | 44.4 | 50.0 | 61.1 | 3.2 | 50.0 | 3.2 | 55.6 | 3.2 | 59.4 |
| PO assays | | | | | | | | | | | | | |
| $10^{-21}$ nTMT vs. solvent | 72.2 | 66.7 | 61.1 | 61.1 | 72.2 | 61.1 | 66.7 | 3.2 | 64.8 | 3.7 | 65.7 | 2.2 | 59.4 |

**Table ST1.** COCRs of WT and ΔD mice in a Y-maze for serial 100-fold diluted odourants (continued).

| Odourant pairs | COCRs of individual ΔD mice (%) | | | | | | avg. of 3 mice (ΔD1-3) | S.E.M. | avg. of 3 mice (ΔD4-6) | S.E.M. | avg. of 6 ΔD mice | S.E.M. | COCR for $P$ = 0.05 |
|---|---|---|---|---|---|---|---|---|---|---|---|---|---|
| | ΔD1 | ΔD2 | ΔD3 | ΔD4 | ΔD5 | ΔD6 | | | | | | | |
| (-)-wnl vs. solvent | | | | | | | | | | | | | |
| $10^{-3}$ (-)-wnl vs. solvent | 66.7 | 87.5 | 91.7 | 61.1 | 61.1 | 61.1 | 81.9 | 7.7 | 61.1 | 0.0 | 71.5 | 5.8 | 59.4 |
| $10^{-5}$ (-)-wnl vs. solvent | 61.1 | 83.3 | 77.8 | 61.1 | 61.1 | 61.1 | 74.1 | 6.7 | 61.1 | 0.0 | 67.6 | 4.2 | 59.4 |
| $10^{-7}$ (-)-wnl vs. solvent | 77.8 | 66.7 | 66.7 | 75.0 | 91.7 | 83.3 | 70.4 | 3.7 | 83.3 | 4.8 | 76.9 | 4.0 | 59.4 |
| $10^{-9}$ (-)-wnl vs. solvent | 77.8 | 66.7 | 77.8 | 72.2 | 72.2 | 83.3 | 74.1 | 3.7 | 75.9 | 3.7 | 75.0 | 2.4 | 59.4 |
| $10^{-11}$ (-)-wnl vs. solvent | 83.3 | 77.8 | 77.8 | 77.8 | 72.2 | 66.7 | 79.6 | 1.9 | 72.2 | 3.2 | 75.9 | 2.3 | 59.4 |
| $10^{-13}$ (-)-wnl vs. solvent | 66.7 | 72.2 | 72.2 | 72.2 | 66.7 | 61.1 | 70.4 | 1.9 | 66.7 | 3.2 | 68.5 | 1.9 | 59.4 |
| $10^{-15}$ (-)-wnl vs. solvent | 61.1 | 66.7 | 61.1 | 66.7 | 61.1 | 66.7 | 63.0 | 1.9 | 64.8 | 1.9 | 63.9 | 1.2 | 59.4 |
| $10^{-17}$ (-)-wnl vs. solvent | 66.7 | 61.1 | 50.0 | 66.7 | 61.1 | 66.7 | 59.3 | 4.9 | 64.8 | 1.9 | 62.0 | 2.7 | 59.4 |
| $10^{-19}$ (-)-wnl vs. solvent | 55.6 | 55.6 | 72.2 | 55.6 | 61.1 | 66.7 | 61.1 | 5.6 | 61.1 | 3.2 | 61.1 | 2.9 | 59.4 |
| $10^{-21}$ (-)-wnl vs. solvent | 50.0 | 50.0 | 33.3 | 38.9 | 50.0 | 61.1 | 44.4 | 5.6 | 50.0 | 6.4 | 47.2 | 4.0 | 59.4 |
| $10^{-23}$ (-)-wnl vs. solvent | 38.9 | 55.6 | 44.4 | 38.9 | 55.6 | 50.0 | 46.3 | 4.9 | 48.1 | 4.9 | 47.2 | 3.1 | 59.4 |
| | | | | | | | | | | | | | |
| PO assays | | | | | | | | | | | | | |
| $10^{-13}$ (-)-wnl vs. solvent | 72.2 | 66.7 | 77.8 | 77.8 | 66.7 | 61.1 | 72.2 | 3.2 | 68.5 | 4.9 | 71.3 | 3.3 | 59.4 |
| $10^{-13}$ (-)-wnl vs. (-)-wnl | 50.0 | 50.0 | 38.9 | 61.1 | 50.0 | 50.0 | 46.3 | 3.7 | 53.7 | 3.7 | 50.0 | 2.5 | 59.4 |
| (+)-wnl vs. solvent | | | | | | | | | | | | | |
| $10^{-3}$ (+)-wnl vs. solvent | 72.2 | 61.1 | 50.0 | 55.6 | 61.1 | 66.7 | 61.1 | 6.4 | 61.1 | 3.2 | 61.1 | 3.2 | 59.4 |
| $10^{-5}$ (+)-wnl vs. solvent | 72.2 | 72.2 | 72.2 | 66.7 | 72.2 | 72.2 | 72.2 | 0.0 | 70.4 | 1.9 | 71.3 | 0.9 | 59.4 |
| $10^{-7}$ (+)-wnl vs. solvent | 66.7 | 66.7 | 83.3 | 66.7 | 66.7 | 55.6 | 72.2 | 5.6 | 63.0 | 3.7 | 67.6 | 3.6 | 59.4 |
| $10^{-9}$ (+)-wnl vs. solvent | 72.2 | 72.2 | 72.2 | 66.7 | 66.7 | 72.2 | 72.2 | 0.0 | 68.5 | 1.9 | 70.4 | 1.2 | 59.4 |
| $10^{-11}$ (+)-wnl vs. solvent | 77.8 | 66.7 | 77.8 | 72.2 | 72.2 | 72.2 | 74.1 | 3.7 | 72.2 | 0.0 | 73.1 | 1.7 | 59.4 |
| $10^{-13}$ (+)-wnl vs. solvent | 55.6 | 55.6 | 55.6 | 61.1 | 44.4 | 38.9 | 55.6 | 0.0 | 48.1 | 6.7 | 51.9 | 3.4 | 59.4 |
| $10^{-15}$ (+)-wnl vs. solvent | 61.1 | 61.1 | 61.1 | 77.8 | 44.4 | 50.0 | 61.1 | 0.0 | 57.4 | 10.3 | 59.3 | 4.7 | 59.4 |
| $10^{-17}$ (+)-wnl vs. solvent | 44.4 | 61.1 | 44.4 | 61.1 | 44.4 | 50.0 | 50.0 | 5.6 | 51.9 | 4.9 | 50.9 | 3.3 | 59.4 |
| | | | | | | | | | | | | | |
| | | | | | | | | | | | | | |
| | | | | | | | | | | | | | |
| PO assays | | | | | | | | | | | | | |
| $10^{-11}$ (+)-wnl vs. solvent | 77.8 | 66.7 | 77.8 | 72.2 | 72.2 | 72.2 | 74.1 | 3.7 | 72.2 | 0.0 | 73.1 | 1.7 | 59.4 |
| $10^{-9}$ (+)-wnl vs. (+)-wnl | 61.1 | 55.6 | 33.3 | 55.6 | 50.0 | 44.4 | 50.0 | 8.5 | 50.0 | 3.2 | 50.0 | 4.1 | 59.4 |
| (-)-wnl vs. (+)-wnl | | | | | | | | | | | | | |
| $10^{-3}$ (-)-wnl vs. (+)-wnl | 27.8 | 50.0 | 55.6 | 61.1 | 38.9 | 44.4 | 44.4 | 8.5 | 48.1 | 6.7 | 46.3 | 4.9 | 59.4 |
| $10^{-5}$ (-)-wnl vs. (+)-wnl | 38.9 | 33.3 | 38.9 | 38.9 | 55.6 | 44.4 | 37.0 | 1.9 | 46.3 | 4.9 | 41.7 | 3.1 | 59.4 |
| $10^{-7}$ (-)-wnl vs. (+)-wnl | 55.6 | 44.4 | 66.7 | 44.4 | 33.3 | 44.4 | 55.6 | 6.4 | 40.7 | 3.7 | 48.1 | 4.7 | 59.4 |
| $10^{-9}$ (-)-wnl vs. (+)-wnl | 38.9 | 66.7 | 55.6 | 38.9 | 44.4 | 50.0 | 53.7 | 8.1 | 41.7 | 2.8 | 48.9 | 5.4 | 59.4 |
| $10^{-11}$ (-)-wnl vs. (+)-wnl | 55.6 | 61.1 | 50.0 | 33.3 | 55.6 | 38.9 | 55.6 | 3.2 | 42.6 | 6.7 | 49.1 | 4.4 | 59.4 |
| $10^{-13}$ (-)-wnl vs. (+)-wnl | 66.7 | 50.0 | 61.1 | 61.1 | 55.6 | 50.0 | 59.3 | 4.9 | 55.6 | 3.2 | 57.4 | 2.7 | 59.4 |
| | | | | | | | | | | | | | |
| | | | | | | | | | | | | | |
| | | | | | | | | | | | | | |
| | | | | | | | | | | | | | |
| | | | | | | | | | | | | | |
| PO assays | | | | | | | | | | | | | |
| $10^{-13}$ (-)-wnl vs. (+)-wnl | 54.2 | 54.2 | 54.2 | 62.5 | 54.2 | 54.2 | 54.2 | 0.0 | 56.9 | 2.8 | 55.6 | 1.4 | 59.4 |
| $10^{-13}$ (-)-wnl vs. solvent | 70.8 | 70.8 | 66.7 | 66.7 | 66.7 | 70.8 | 69.4 | 1.4 | 68.1 | 1.4 | 68.8 | 0.9 | 59.4 |
| (-)-car vs. solvent | | | | | | | | | | | | | |
| $10^{-3}$ (-)-car vs. solvent | 66.7 | 77.8 | 77.8 | 66.7 | 72.2 | 66.7 | 74.1 | 3.7 | 68.5 | 1.9 | 71.3 | 2.2 | 59.4 |
| $10^{-5}$ (-)-car vs. solvent | 55.6 | 77.8 | 83.3 | 83.3 | 83.3 | 72.2 | 72.2 | 8.5 | 79.6 | 3.7 | 75.9 | 4.5 | 59.4 |
| $10^{-7}$ (-)-car vs. solvent | 72.2 | 83.3 | 77.8 | 72.2 | 66.7 | 66.7 | 77.8 | 3.2 | 68.5 | 1.9 | 73.1 | 2.7 | 59.4 |
| $10^{-9}$ (-)-car vs. solvent | 77.8 | 77.8 | 83.3 | 77.8 | 61.1 | 72.2 | 79.6 | 1.9 | 70.4 | 4.9 | 75.0 | 3.1 | 59.4 |
| $10^{-11}$ (-)-car vs. solvent | 83.3 | 72.2 | 88.9 | 94.4 | 72.2 | 72.2 | 81.5 | 4.9 | 79.6 | 7.4 | 80.6 | 4.0 | 59.4 |
| $10^{-13}$ (-)-car vs. solvent | 83.3 | 77.8 | 66.7 | 83.3 | 72.2 | 72.2 | 75.9 | 4.9 | 75.9 | 3.7 | 75.9 | 2.7 | 59.4 |
| $10^{-15}$ (-)-car vs. solvent | 61.1 | 66.7 | 66.7 | 66.7 | 72.2 | 61.1 | 64.8 | 1.9 | 66.7 | 3.2 | 65.7 | 1.7 | 59.4 |
| $10^{-17}$ (-)-car vs. solvent | 77.8 | 61.1 | 72.2 | 72.2 | 55.6 | 66.7 | 70.4 | 4.9 | 64.8 | 4.9 | 67.6 | 3.3 | 59.4 |
| $10^{-19}$ (-)-car vs. solvent | 61.1 | 55.6 | 50.0 | 61.1 | 72.2 | 50.0 | 55.6 | 3.2 | 61.1 | 6.4 | 58.3 | 3.4 | 59.4 |
| | | | | | | | | | | | | | |
| | | | | | | | | | | | | | |
| | | | | | | | | | | | | | |
| PO assays | | | | | | | | | | | | | |
| $10^{-9}$ (-)-car vs. solvent | 83.3 | 66.7 | 72.2 | 66.7 | 72.2 | 66.7 | 74.1 | 4.9 | 68.5 | 1.9 | 71.3 | 2.7 | 59.4 |
| $10^{-9}$ (-)-car vs. (-)-car | 55.6 | 55.6 | 55.6 | 55.6 | 55.6 | 38.9 | 55.6 | 0.0 | 50.0 | 5.6 | 52.8 | 2.8 | 59.4 |

**Table ST1.** COCRs of WT and ΔD mice in a Y-maze for serial 100-fold diluted odourants (continued).

| Odourant pairs | COCRs of individual ΔD mice (%) | | | | | | avg. of 3 mice (ΔD1-3) | S.E.M. | avg. of 3 mice (ΔD4-6) | S.E.M. | avg. of 6 ΔD mice | S.E.M. | COCR for $P$ = 0.05 |
|---|---|---|---|---|---|---|---|---|---|---|---|---|---|
| | ΔD1 | ΔD2 | ΔD3 | ΔD4 | ΔD5 | ΔD6 | | | | | | | |
| (+)-car vs. solvent | | | | | | | | | | | | | |
| $10^{-3}$ (+)-car vs. solvent | 72.2 | 72.2 | 72.2 | 61.1 | 72.2 | 61.1 | 72.2 | 0.0 | 64.8 | 3.7 | 68.5 | 2.3 | 59.4 |
| $10^{-5}$ (+)-car vs. solvent | 61.1 | 72.2 | 72.2 | 55.6 | 55.6 | 61.1 | 68.5 | 3.7 | 57.4 | 1.9 | 63.0 | 3.1 | 59.4 |
| $10^{-7}$ (+)-car vs. solvent | 77.8 | 77.8 | 77.8 | 77.8 | 72.2 | 66.7 | 77.8 | 0.0 | 72.2 | 3.2 | 75.0 | 1.9 | 59.4 |
| $10^{-9}$ (+)-car vs. solvent | 72.2 | 77.8 | 61.1 | 72.2 | 66.7 | 61.1 | 70.4 | 4.9 | 66.7 | 3.2 | 68.5 | 2.7 | 59.4 |
| $10^{-11}$ (+)-car vs. solvent | 72.2 | 83.3 | 72.2 | 66.7 | 66.7 | 61.1 | 75.9 | 3.7 | 64.8 | 1.9 | 70.4 | 3.1 | 59.4 |
| $10^{-13}$ (+)-car vs. solvent | 72.2 | 72.2 | 61.1 | 61.1 | 61.1 | 61.1 | 68.5 | 3.7 | 61.1 | 0.0 | 64.8 | 2.3 | 59.4 |
| $10^{-15}$ (+)-car vs. solvent | 66.7 | 50.0 | 55.6 | 66.7 | 55.6 | 55.6 | 57.4 | 4.9 | 59.3 | 3.7 | 58.3 | 2.8 | 59.4 |
| | | | | | | | | | | | | | |
| | | | | | | | | | | | | | |
| | | | | | | | | | | | | | |
| | | | | | | | | | | | | | |
| PO assays | | | | | | | | | | | | | |
| $10^{-11}$ (+)-car vs. solvent | 61.1 | 66.7 | 66.7 | 66.7 | 66.7 | 66.7 | 64.8 | 1.9 | 66.7 | 0.0 | 65.7 | 0.9 | 59.4 |
| (-)-car vs. (+)-car | | | | | | | | | | | | | |
| $10^{-3}$ (-)-car vs. (+)-car | 61.1 | 61.1 | 55.6 | 66.7 | 55.6 | 61.1 | 59.3 | 1.9 | 61.1 | 3.2 | 60.2 | 1.7 | 59.4 |
| $10^{-5}$ (-)-car vs. (+)-car | 61.1 | 72.2 | 66.7 | 72.2 | 55.6 | 61.1 | 66.7 | 3.2 | 63.0 | 4.9 | 64.8 | 2.7 | 59.4 |
| $10^{-7}$ (-)-car vs. (+)-car | 72.2 | 61.1 | 61.1 | 66.7 | 55.6 | 55.6 | 64.8 | 3.7 | 59.3 | 3.7 | 62.0 | 2.7 | 59.4 |
| $10^{-9}$ (-)-car vs. (+)-car | 55.6 | 72.2 | 61.1 | 72.2 | 55.6 | 55.6 | 63.0 | 4.9 | 61.1 | 5.6 | 62.0 | 3.3 | 59.4 |
| $10^{-11}$ (-)-car vs. (+)-car | 50.0 | 55.6 | 66.7 | 50.0 | 44.4 | 44.4 | 57.4 | 4.9 | 46.3 | 1.9 | 51.9 | 3.4 | 59.4 |
| $10^{-13}$ (-)-car vs. (+)-car | 61.1 | 55.6 | 55.6 | 61.1 | 66.7 | 50.0 | 57.4 | 1.9 | 59.3 | 4.9 | 58.3 | 2.4 | 59.4 |
| $10^{-15}$ (-)-car vs. (+)-car | 55.6 | 50.0 | 61.1 | 61.1 | 55.6 | 50.0 | 55.6 | 3.2 | 55.6 | 3.2 | 55.6 | 2.0 | 59.4 |
| $10^{-17}$ (-)-car vs. (+)-car | 50.0 | 55.6 | 61.1 | 61.1 | 61.1 | 50.0 | 55.6 | 3.2 | 57.4 | 3.7 | 56.5 | 2.2 | 59.4 |
| $10^{-19}$ (-)-car vs. (+)-car | 50.0 | 55.6 | 66.7 | 55.6 | 61.1 | 50.0 | 53.7 | 1.9 | 55.6 | 3.2 | 54.6 | 1.7 | 59.4 |
| | | | | | | | | | | | | | |
| | | | | | | | | | | | | | |
| PO assays | | | | | | | | | | | | | |
| $10^{-11}$ (-)-car vs. solvent | 77.8 | 72.2 | 77.8 | 72.2 | 83.3 | 66.7 | 75.9 | 1.9 | 74.1 | 4.9 | 75.0 | 2.4 | 59.4 |
| nTMT vs dpg | | | | | | | | | | | | | |
| $10^{-7}$ nTMT vs. solvent | 61.1 | 61.1 | 66.7 | 66.7 | 61.1 | 66.7 | 63.0 | 1.9 | 64.8 | 1.9 | 63.9 | 1.2 | 59.4 |
| $10^{-9}$ nTMT vs. solvent | 83.3 | 72.2 | 66.7 | 61.1 | 66.7 | 88.9 | 74.1 | 4.9 | 72.2 | 8.5 | 73.1 | 4.4 | 59.4 |
| $10^{-11}$ nTMT vs. solvent | 77.8 | 72.2 | 77.8 | 72.2 | 72.2 | 88.9 | 75.9 | 1.9 | 77.8 | 5.6 | 76.9 | 2.7 | 59.4 |
| $10^{-13}$ nTMT vs. solvent | 77.8 | 94.4 | 83.3 | 77.8 | 83.3 | 72.2 | 85.2 | 4.9 | 77.8 | 3.2 | 81.5 | 3.1 | 59.4 |
| $10^{-15}$ nTMT vs. solvent | 77.8 | 66.7 | 83.3 | 77.8 | 72.2 | 66.7 | 75.9 | 4.9 | 72.2 | 3.2 | 74.1 | 2.7 | 59.4 |
| $10^{-17}$ nTMT vs. solvent | 77.8 | 88.9 | 83.3 | 55.6 | 50.0 | 61.1 | 83.3 | 3.2 | 55.6 | 3.2 | 69.4 | 6.5 | 59.4 |
| $10^{-19}$ nTMT vs. solvent | 77.8 | 55.6 | 72.2 | 50.0 | 55.6 | 50.0 | 68.5 | 6.7 | 51.9 | 1.9 | 60.2 | 4.8 | 59.4 |
| $10^{-21}$ nTMT vs. solvent | 44.4 | 61.1 | 50.0 | 50.0 | 55.6 | 50.0 | 51.9 | 4.9 | 51.9 | 1.9 | 51.9 | 2.3 | 59.4 |
| | | | | | | | | | | | | | |
| PO assays | | | | | | | | | | | | | |
| $10^{-17}$ nTMT vs. solvent | 61.1 | 83.3 | 77.8 | 61.1 | 66.7 | 83.3 | 74.1 | 6.7 | 70.4 | 6.7 | 72.2 | 4.3 | 59.4 |

**Table ST1.** COCRs of WT and ΔD mice in a Y-maze for serial 100-fold diluted odourants (continued).

| Odourant concentration | Odourants | | | | | | | | | | |
|---|---|---|---|---|---|---|---|---|---|---|---|
| | (–)-wnl | | | | | Total of target-best ORs | (+)-wnl | | | | |
| | Tuning classes of OSNs/ORs | | | OR overlap | The largest class | | The largest class | OR overlap | Tuning classes of OSNs/ORs | | |
| | (–)-wnl* | (–)-/(+)-wnl | (+)-wnl | | | | | | (–)-wnl | (–)-/(+)-wnl | (+)-wnl* |
| 10 nM | 1 (1:0) 50% | 1 (1:0) 50% | 0 (0:0) 0% | 50% (1/2) | (–), (–)/(+) | > | (–)/(+) | 100% (1/1) | 0 (0:0) 0% | 1 (1:0) 100% | 0 (0:0) 0% |
| 100 nM | 1 (1:0) 50% | 1 (1:0) 50% | 0 (0:0) 0% | 50% (1/2) | (–), (–)/(+) | > | (–)/(+) | 100% (1/1) | 0 (0:0) 0% | 1 (1:0) 100% | 0 (0:0) 0% |
| 1 μM | 3 (2:1) 75% | 1 (1:0) 25% | 0 (0:0) 0% | 25% (1/4) | (–) | > | (–)/(+), (+) | 50% (1/2) | 0 (0:0) 0% | 1 (1:0) 50% | 1 (1:0) 50% |
| 10 μM | 14 (7:7) 87.5% | 2 (2:0) 12.5% | 0 (0:0) 0% | 25% (4/16) | (–) | > | (–)/(+), (–) | 80% (4/5) | 2 (2:0) 40% | 2 (2:0) 40% | 1 (1:0) 20% |
| 100 μM | 38 (21:17) 84% | 3 (3:0) 7% | 4 (2:2) 9% | 42% (19/45) | (–) | > | (–) | 95% (19/20) | 12 (6:6) 60% | 3 (3:0) 15% | 5 (3:2) 25% |

**Table ST2.** Dose-dependent subpopulations of wine lactone-responsive OSNs/ORs. Of 1,746 olfactory sensory neurons (OSNs) (1,207 dorsal zone: 539 ventral zone) assayed by $Ca^{2+}$ imaging, 2, 5, 17 and 46 OSNs/ORs responded to either or both wine lactone enantiomers at 10-100 nM, 1, 10, 100 μM, respectively. They were classified by their tuning to target enantiomers. Target-best OSNs/ORs* are those more sensitive to the target enantiomer than to non-target enantiomer. Percentage of each class responsive to the target at a given concentration is listed in the lower row. OR overlap indicates percentage (Numbers) of overlapping OSN/OR in enantiomer OR codes at each concentration. (–)-wnl, (–)-wine lactone; (+)-wnl, (+)-wine lactone.

| Odourant conc. | Odourants | | | | | | | | | | | | |
|---|---|---|---|---|---|---|---|---|---|---|---|---|---|
| | (–)-car | | | | | | Total of target-best ORs | (+)-car | | | | | |
| | Tuning classes of OSNs/ORs | | | | OR overlap | The largest class | | The largest class | OR overlap | Tuning classes of OSNs/ORs | | | |
| | (–)-car* | (–)-car /others | (–)-/(+)-car | (+)-car | | | | | | (–)-car | (–)-/(+)-car | (+)-car /others | (+)-car* |
| 1 μM | 4 (2:2) 45% | 2 (1:1) 22% | 3 (2:1) 33% | 0 (0:0) 0% | 55% (5/9) | (–) | > | (–)/(+), (+)‡ | 55% (5/9) | 2 (0:2) 22%† | 3 (2:1) 33% | 2 (1:1) 22% | 2(1.5:0.5) 22%(11) |
| 10 μM | 18(7.5:10.5) 25.5%(7) | 6 (2:4) 8.5%(3) | 44 (20:24) 62%(5.5) | 3 (2.5:0.5) 4%(1.5) | 79% (56/71) | (–)/(+) | = | (–)/(+) | 62% (51/82) | 9 (4:5) 11%(5) | 44 (20:24) 53.5%(5) | 8 (2.5:5.5) 10%(3.5) | 21(5.5:15.5) 25.5%(3.5) |
| | Identified carvone-responsive ORs and their sensitivities to carvone enantiomers | | | | | | | | | | | | |
| 1 μM | *car-c5* (+)car(10) | | | | | | | | | | | | |
| | | | *car-n266* less | | | | | | | | *car-n266* greater | | |
| | | | *car-b85* greater | | | | | | | | *car-b85* less | | |
| | | | *car-n272* greater | | | | | | | | *car-n272* less | | |
| 10 μM | *car-c10* (+)car(100) | | | | | | | | | | | | |
| | | | | | | | | | | | | | *car-b158* (–)car(100) |
| | | | *car-b130* greater | | | | | | | | *car-b130* less | | |
| | | | *car-n270* greater | | | | | | | | *car-n270* less | | |
| | *car-257* (+)car(100) | | | | | | | | | | | | |
| | | | *car-n271* equal | | | | | | | | *car-n271* equal | | |
| 100 μM | *car-c260* | | | | | | | | | | | | |
| | | | | | | | | | | | | | *car-c258* |
| | | | *car-c6* equal | | | | | | | | *car-c6* equal | | |
| | | | *car-c255* less | | | | | | | | *car-c255* greater | | |
| | *car-c15* | | | | | | | | | | | | |

**Table ST3.** Dose-dependent subpopulations of carvone-responsive OSNs/ORs. Of 2,740 OSNs/ORs assayed by $Ca^{2+}$ imaging, 13 and 89 OSNs/ORs (dorsal zone: ventral zone) responded to either or both of carvone enantiomers at 1 and 10 μM, respectively, and were classified by their tuning to target odourants. Target-best OSNs/ORs* are those more sensitive to the target enantiomer than to the non-target enantiomer. Percentage of each class responsive to target at a given concentration is listed in the lower rows. Percentage† indicates OSNs/ORs with response amplitudes less than the half of the others. The percentages in parentheses indicate OSNs/ORs that were prepared from uncertain zones and counted in each zone by a factor of 1/2. OR overlap indicates percentage (numbers) of overlapping OSN/OR in enantiomer OR codes at each concentration. The largest class‡ is the sum of (+)-car and (+)-car/others OSNs/ORs. In the lower part, relative response amplitudes of 15 identified carvone ORs are shown with the lowest responsive concentrations (μM) of target (left column) and non-target carvone in parentheses (see Fig. 3).

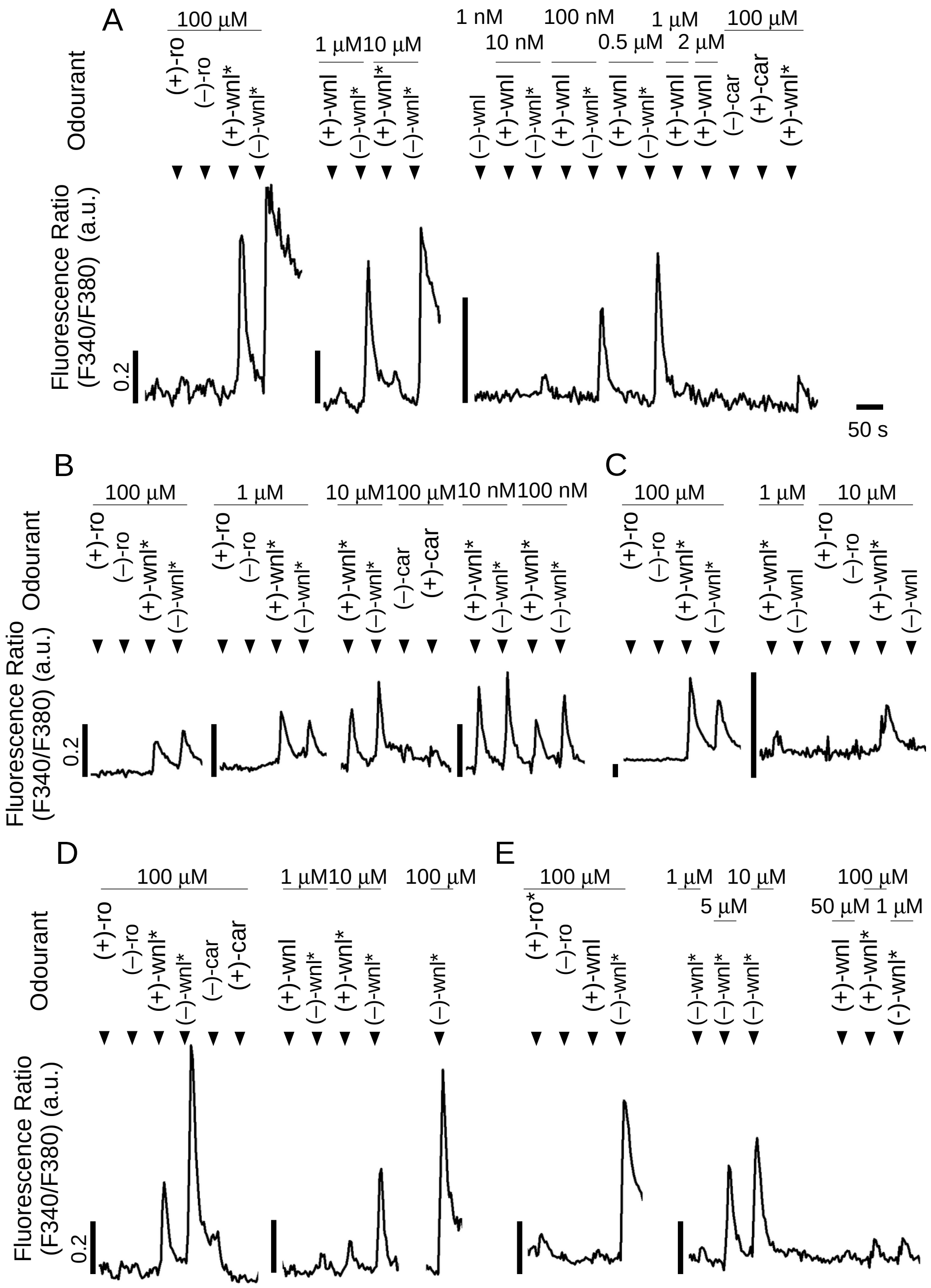


**Figure S2.** Odour responses of the most sensitive OSNs for wine lactones in mice. Odour responses were measured using intracellular $Ca^{2+}$-imaging. A, OSN tuned to 10 nM (–)-wnl . B, OSN tuned to 10 nM (–)-wnl and (+)-wnl. C, OSN tuned to 1 μM (+)-wnl. D and E, OSNs tuned to 1 μM (–)-wnl. (–)-ro: (2*S*,4*R*)- (–)-cis-rose oxide; (+)-ro:(2*R*,4*S*)-(+)-cis-rose oxide. The arrows indicate the time at which odourant solutions of the indicated concentrations were applied to the OSNs for 4 s. The odourants that evoked characteristic odour responses are indicated by the asterisks. All OSNs, except for that shown in E, were obtained from the dorsal zone.

| Odourant pairs | significance of difference between 2 strains | | WT mice | | ΔD mice | |
|---|---|---|---|---|---|---|
| | F value | *P* value | COCR (%) (n = 6) | S.E.M. | COCR (%) (n = 6) | S.E.M. |
| (-)-wnl vs. solvent | | | | | | |
| $10^{-3}$ (-)-wnl vs. solvent | 4.13 | $7.0 \times 10^{-2}$ | 87.0 | 4.7 | 71.5 | 5.8 |
| $10^{-5}$ (-)-wnl vs. solvent | 5.52 | $4.1 \times 10^{-2}$ | 77.8 | 1.4 | 67.6 | 4.2 |
| $10^{-7}$ (-)-wnl vs. solvent | 0.61 | 0.45 | 72.2 | 4.5 | 76.9 | 4.0 |
| $10^{-9}$ (-)-wnl vs. solvent | 0.07 | 0.80 | 74.1 | 3.1 | 75.0 | 2.4 |
| $10^{-11}$ (-)-wnl vs. solvent | 0.58 | 0.46 | 79.6 | 4.2 | 75.9 | 2.3 |
| $10^{-13}$ (-)-wnl vs. solvent | 5.35 | $4.3 \times 10^{-2}$ | 79.6 | 4.5 | 68.5 | 1.9 |
| $10^{-15}$ (-)-wnl vs. solvent | 47.57 | $4.2 \times 10^{-5}$ | 79.6 | 1.9 | 63.9 | 1.2 |
| $10^{-17}$ (-)-wnl vs. solvent | 12.86 | $5.0 \times 10^{-3}$ | 75.9 | 2.7 | 62.0 | 2.7 |
| $10^{-19}$ (-)-wnl vs. solvent | 5.18 | $4.6 \times 10^{-2}$ | 71.3 | 3.3 | 61.1 | 2.9 |
| $10^{-21}$ (-)-wnl vs. solvent | 18.06 | $1.7 \times 10^{-3}$ | 67.6 | 2.7 | 47.2 | 4.0 |
| $10^{-23}$ (-)-wnl vs. solvent | 6.86 | $2.6 \times 10^{-2}$ | 59.3 | 3.1 | 47.2 | 3.1 |
| (+)-wnl vs. solvent | | | | | | |
| $10^{-3}$ (+)-wnl vs. solvent | 9.79 | $1.1 \times 10^{-2}$ | 76.6 | 3.9 | 61.1 | 3.2 |
| $10^{-5}$ (+)-wnl vs. solvent | 2.92 | 0.12 | 77.8 | 3.5 | 71.3 | 0.9 |
| $10^{-7}$ (+)-wnl vs. solvent | 5.08 | $4.8 \times 10^{-2}$ | 75.7 | 4.2 | 67.6 | 3.6 |
| $10^{-9}$ (+)-wnl vs. solvent | 21.00 | $1.0 \times 10^{-3}$ | 83.1 | 3.1 | 70.4 | 1.2 |
| $10^{-11}$ (+)-wnl vs. solvent | 0.09 | 0.76 | 74.5 | 4.1 | 73.1 | 1.7 |
| $10^{-13}$ (+)-wnl vs. solvent | 54.55 | $2.4 \times 10^{-5}$ | 82.9 | 2.3 | 51.9 | 3.4 |
| $10^{-15}$ (+)-wnl vs. solvent | 14.72 | $3.3 \times 10^{-3}$ | 75.2 | 3.1 | 59.3 | 4.7 |
| $10^{-17}$ (+)-wnl vs. solvent | 31.87 | $2.1 \times 10^{-4}$ | 75.2 | 2.8 | 50.9 | 3.3 |
| (-)-wnl vs. (+)-wnl | | | | | | |
| $10^{-3}$ (-)-wnl vs. (+)-wnl | 15.37 | $2.9 \times 10^{-3}$ | 69.4 | 3.4 | 46.3 | 4.9 |
| $10^{-5}$ (-)-wnl vs. (+)-wnl | 6.36 | $3.0 \times 10^{-2}$ | 54.3 | 3.9 | 41.7 | 3.1 |
| $10^{-7}$ (-)-wnl vs. (+)-wnl | 11.45 | $7.0 \times 10^{-3}$ | 73.1 | 5.8 | 48.1 | 4.7 |
| $10^{-9}$ (-)-wnl vs. (+)-wnl | 13.54 | $4.3 \times 10^{-3}$ | 68.5 | 2.7 | 48.9 | 5.4 |
| $10^{-11}$ (-)-wnl vs. (+)-wnl | 4.78 | $5.4 \times 10^{-2}$ | 62.0 | 3.9 | 49.1 | 4.4 |
| $10^{-13}$ (-)-wnl vs. (+)-wnl | 6.01 | $3.4 \times 10^{-2}$ | 72.2 | 5.4 | 57.4 | 2.7 |

**Table ST4.** Significance of the COCR differences between WT and ΔD mice.

| Odourant pairs | significance of difference between 2 strains | | WT mice | | ΔD mice | |
|---|---|---|---|---|---|---|
| | F value | *P* value | COCR (%) (n = 6) | S.E.M. | COCR (%) (n = 6) | S.E.M. |
| (-)-car vs. solvent | | | | | | |
| $10^{-3}$ (-)-car vs. solvent | 4.24 | $6.7 \times 10^{-2}$ | 81.5 | 4.2 | 71.3 | 2.2 |
| $10^{-5}$ (-)-car vs. solvent | 1.45 | 0.26 | 82.4 | 3.0 | 75.9 | 4.5 |
| $10^{-7}$ (-)-car vs. solvent | 0.03 | 0.87 | 72.5 | 2.9 | 73.1 | 2.7 |
| $10^{-9}$ (-)-car vs. solvent | 2.21 | 0.17 | 67.6 | 3.9 | 75.0 | 3.1 |
| $10^{-11}$ (-)-car vs. solvent | 0.41 | 0.54 | 75.9 | 6.2 | 80.6 | 4.0 |
| $10^{-13}$ (-)-car vs. solvent | 0.00 | 1.00 | 75.9 | 5.3 | 75.9 | 2.7 |
| $10^{-15}$ (-)-car vs. solvent | 3.70 | 0.08 | 72.2 | 2.9 | 65.7 | 1.7 |
| $10^{-17}$ (-)-car vs. solvent | 1.58 | 0.24 | 74.1 | 4.0 | 67.6 | 3.3 |
| $10^{-19}$ (-)-car vs. solvent | 5.14 | $4.7 \times 10^{-2}$ | 67.6 | 2.2 | 58.3 | 3.4 |
| (+)-car vs. solvent | | | | | | |
| $10^{-3}$ (+)-car vs. solvent | 6.43 | $3.0 \times 10^{-2}$ | 79.6 | 3.7 | 68.5 | 2.3 |
| $10^{-5}$ (+)-car vs. solvent | 12.34 | $5.6 \times 10^{-3}$ | 79.6 | 3.7 | 63.0 | 3.1 |
| $10^{-7}$ (+)-car vs. solvent | 2.22 | 0.17 | 79.6 | 2.3 | 75.0 | 1.9 |
| $10^{-9}$ (+)-car vs. solvent | 1.21 | 0.30 | 64.8 | 1.9 | 68.5 | 2.7 |
| $10^{-11}$ (+)-car vs. solvent | 0.07 | 0.79 | 71.3 | 3.0 | 70.4 | 3.1 |
| $10^{-13}$ (+)-car vs. solvent | 4.49 | $6.0 \times 10^{-2}$ | 72.2 | 2.9 | 64.8 | 2.3 |
| $10^{-15}$ (+)-car vs. solvent | 7.67 | $2.0 \times 10^{-2}$ | 72.2 | 4.1 | 58.3 | 2.8 |
| (-)-car vs. (+)-car | | | | | | |
| $10^{-3}$ (-)-car vs. (+)-car | 28.13 | $3.5 \times 10^{-4}$ | 84.3 | 4.2 | 60.2 | 1.7 |
| $10^{-5}$ (-)-car vs. (+)-car | 8.55 | $1.5 \times 10^{-2}$ | 77.8 | 3.5 | 64.8 | 2.7 |
| $10^{-7}$ (-)-car vs. (+)-car | 9.13 | $1.3 \times 10^{-2}$ | 73.1 | 2.7 | 62.0 | 2.7 |
| $10^{-9}$ (-)-car vs. (+)-car | 1.60 | 0.23 | 68.5 | 3.7 | 62.0 | 3.3 |
| $10^{-11}$ (-)-car vs. (+)-car | 27.17 | $3.9 \times 10^{-4}$ | 75.9 | 3.1 | 51.9 | 3.4 |
| $10^{-13}$ (-)-car vs. (+)-car | 2.43 | 0.15 | 64.8 | 3.4 | 58.3 | 2.4 |
| $10^{-15}$ (-)-car vs. (+)-car | 10.61 | $8.6 \times 10^{-3}$ | 66.7 | 2.9 | 55.6 | 2.0 |
| $10^{-17}$ (-)-car vs. (+)-car | 17.90 | $1.7 \times 10^{-3}$ | 75.0 | 3.7 | 56.5 | 2.2 |
| $10^{-19}$ (-)-car vs. (+)-car | 8.43 | $1.6 \times 10^{-2}$ | 67.6 | 2.7 | 54.6 | 1.7 |
| nTMT vs. solvent | | | | | | |
| $10^{-9}$ nTMT vs. solvent | 0.40 | 0.54 | 68.5 | 6.0 | 73.1 | 4.4 |
| $10^{-11}$ nTMT vs. solvent | 5.02 | $4.9 \times 10^{-2}$ | 67.6 | 3.0 | 76.9 | 2.7 |
| $10^{-13}$ nTMT vs. solvent | 0.30 | 0.59 | 78.7 | 4.2 | 81.5 | 3.1 |
| $10^{-15}$ nTMT vs. solvent | 0.09 | 0.77 | 75.9 | 4.9 | 74.1 | 2.7 |
| $10^{-17}$ nTMT vs. solvent | 0.01 | 0.91 | 68.5 | 3.7 | 69.4 | 6.5 |
| $10^{-19}$ nTMT vs. solvent | 1.67 | 0.23 | 66.7 | 1.4 | 60.2 | 4.8 |
| $10^{-21}$ nTMT vs. solvent | 3.72 | $8.3 \times 10^{-2}$ | 63.9 | 5.9 | 51.9 | 2.3 |

**Table ST4.** Significance of the COCR differences between WT and ΔD mice (continued).

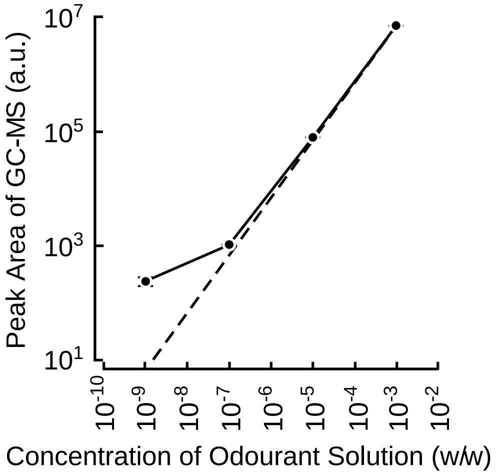


**Figure S3.** Dose-dependency of the peak areas of diluted odourant solutions in gas chromatography-mass spectrometry (GC-MS). The peak area per unit dose seemed too large for the $10^{-9}$-w/w odourant solution compared with those at higher concentrations. This was likely due to background noise. This result suggests that the detection limit of this GC-MS measurement is approximately 10 ppb for (*R*)-(-)-carvone. The theoretical curve (dashed line) is the plot of the peak areas at the concentration ($C_x$) with correction by the term -(peak_area_at_$10^{-9}$) + (peak_area_at_ $10^{-3} \times C_x/10^{-3}$)

| target conc. (w/w) | measured peak area (a.u.) | | | | | dilution factor (D, ratio of peak area per unit dose) | | |
|---|---|---|---|---|---|---|---|---|
| | dilution 1 (D1) | dilution 2 (D2) | dilution 3 (D3) | average ± S.E.M. | | D1 | D2 | D3 |
| | | | | | average | 1.01 | 1.00 | 1.02 |
| $10^{-3}$ | 7,029,438.5 | 6,806,454.0 | 6,703,118.0 | $6.85 \times 10^6$ ± $9.63 \times 10^4$ | | 1.18 | 1.11 | 1.07 |
| $10^{-5}$ | 83,192.0 | 75,972.5 | 71,889.0 | $7.70 \times 10^4$ ± $3.30 \times 10^3$ | | 1.04 | 0.90 | 1.21 |
| $10^{-7}$ | 1,033.5 | 893.5 | 1,181.0 | $1.04 \times 10^3$ ± $8.30 \times 10^1$ | | 0.82 | 1.00 | 0.77 |
| $10^{-9}$ | 178.5 | 219.0 | 321.0 | $2.40 \times 10^2$ ± $4.24 \times 10^1$ | $D_{avg}$ | 1.0109 ± 0.0515 | | |

**Table ST5.** Peak areas in GC-MS and calculation of the dilution factors for the 100-fold dilution series. The dilution factors were obtained as the ratios of peak areas per unit dose between subsequent concentrations. (*R*)-(−)-carvone was used as the standard odourant. The dose-dependency of the peak areas was shown in Supplementary Information Fig. S3.

| Target concentration (C) (w/w) | Number of dilution steps (N) | Accumulated average dilution factor ($A_{avg} = D_{avg}$^N) | Estimated conc. = C × $A_{avg}$ (w/w) | Accumulated maximum dilution factor ($A_{max} = D_{max}$^N) | Estimated maximum conc. = C × $A_{max}$ (w/w) |
|---|---|---|---|---|---|
| $10^{-3}$ | 1 | 1.01 | $1.0 \times 10^{-3}$ | 1.21 | $1.2 \times 10^{-3}$ |
| $10^{-5}$ | 2 | 1.02 | $1.0 \times 10^{-5}$ | 1.47 | $1.5 \times 10^{-5}$ |
| $10^{-7}$ | 3 | 1.03 | $1.0 \times 10^{-7}$ | 1.78 | $1.8 \times 10^{-7}$ |
| $10^{-9}$ | 4 | 1.04 | $1.0 \times 10^{-9}$ | 2.15 | $2.2 \times 10^{-9}$ |
| $10^{-11}$ | 5 | 1.06 | $1.1 \times 10^{-11}$ | 2.60 | $2.6 \times 10^{-11}$ |
| $10^{-13}$ | 6 | 1.07 | $1.1 \times 10^{-13}$ | 3.15 | $3.2 \times 10^{-13}$ |
| $10^{-15}$ | 7 | 1.08 | $1.1 \times 10^{-15}$ | 3.82 | $3.8 \times 10^{-15}$ |
| $10^{-17}$ | 8 | 1.09 | $1.1 \times 10^{-17}$ | 4.62 | $4.6 \times 10^{-17}$ |
| $10^{-19}$ | 9 | 1.10 | $1.1 \times 10^{-19}$ | 5.60 | $5.6 \times 10^{-19}$ |
| $10^{-21}$ | 10 | 1.11 | $1.1 \times 10^{-21}$ | 6.78 | $6.8 \times 10^{-21}$ |
| $10^{-23}$ | 11 | 1.13 | $1.1 \times 10^{-23}$ | 8.21 | $8.2 \times 10^{-23}$ |
| $10^{-25}$ | 12 | 1.14 | $1.1 \times 10^{-25}$ | 9.94 | $9.9 \times 10^{-25}$ |

**Table ST6.** Estimated odourant concentrations for the 100-fold dilution series. Averaged dilution factor, Davg = 1.0109 ± 0.0515, and the maximum dilution factor, $D_{max}$ = 1.21, are shown in Supplementary Information Table ST5.
